# The autism risk factor CHD8 is a chromatin activator in human neurons and functionally dependent on the ERK-MAPK pathway effector ELK1

**DOI:** 10.1038/s41598-022-23614-x

**Published:** 2022-12-27

**Authors:** Bahareh Haddad Derafshi, Tamas Danko, Soham Chanda, Pedro J. Batista, Ulrike Litzenburger, Qian Yi Lee, Yi Han Ng, Anu Sebin, Howard Y. Chang, Thomas C. Südhof, Marius Wernig

**Affiliations:** 1 Institute for Stem Cell Biology and Regenerative Medicine, Stanford University School of Medicine, 265 Campus Drive, Stanford, CA 94305 USA; 2 Department of Molecular and Cellular Physiology, Stanford University School of Medicine, 265 Campus Drive, Stanford, CA 94305 USA; 3 Departments of Pathology and Chemical and Systems Biology, Stanford University School of Medicine, 265 Campus Drive, Stanford, CA 94305 USA; 4 Howard Hughes Medical Institute, 4000 Jones Bridge Road, Chevy Chase, MD 20815-6789 USA; 5 Center for Personal Dynamic Regulomes, Stanford University School of Medicine, 265 Campus Drive, Stanford, CA 94305 USA; 6 Program in Epithelial Biology, Stanford University School of Medicine, 265 Campus Drive, Stanford, CA 94305 USA; 7 Department of Genetics, Stanford University School of Medicine, 265 Campus Drive, Stanford, CA 94305 USA; 8 Present Address: Laboratory of Cell Biology, Center for Cancer Research, National Cancer Institute, National Institutes of Health, Bethesda, MD 20892 USA; 9 Present Address: Celgene, San Francisco, CA 94158 USA

**Keywords:** Epigenetics in the nervous system, Neurodevelopmental disorders, Genetics, Neuroscience, Stem cells, Diseases

## Abstract

The chromodomain helicase DNA-binding protein CHD8 is the most frequently mutated gene in autism spectrum disorder. Despite its prominent disease involvement, little is known about its molecular function in the human brain. CHD8 is a chromatin regulator which binds to the promoters of actively transcribed genes through genomic targeting mechanisms which have yet to be fully defined. By generating a conditional loss-of-function and an endogenously tagged allele in human pluripotent stem cells, we investigated the molecular function and the interaction of CHD8 with chromatin in human neurons. Chromatin accessibility analysis and transcriptional profiling revealed that CHD8 functions as a transcriptional activator at its target genes in human neurons. Furthermore, we found that CHD8 chromatin targeting is cell context-dependent. In human neurons, CHD8 preferentially binds at *ETS* motif-enriched promoters. This enrichment is particularly prominent on the promoters of genes whose expression significantly changes upon the loss of CHD8. Indeed, among the *ETS* transcription factors, we identified ELK1 as being most highly correlated with CHD8 expression in primary human fetal and adult cortical neurons and most highly expressed in our stem cell-derived neurons. Remarkably, ELK1 was necessary to recruit CHD8 specifically to ETS motif-containing sites. These findings imply that ELK1 and CHD8 functionally cooperate to regulate gene expression and chromatin states at MAPK/ERK target genes in human neurons. Our results suggest that the MAPK/ERK/ELK1 axis potentially contributes to the pathogenesis caused by *CHD8 *mutations in human neurodevelopmental disorders.

## Introduction

Large-scale genetic studies of cohorts with autism spectrum disorder (ASD) reveal common at-risk loci associated with a complex and broad range of disease-relevant traits^1–9^. Many attributed ASD risk genes converge on chromatin regulation and synapse function^2,10–15^. Therefore, it is possible that the pathophysiology of ASD follows a regulated developmental pathway initiated by high-risk genes. Among all ASD risk genes known to date CHD8 carries the highest number of recurrent disruptive mutations, exhibiting distinct phenotypic features in human and animal models^3,4,5,16–20^.

Previous studies showed that CHD8 is distinct from other ATP-dependent remodelers in that its heterozygous loss of function in various tissue and cell types caused only mild gene expression changes and no significant phenotypes^21–48^. Conversely, homozygote CHD8 loss-of-function mutations in proliferative cells led to programmed cell death and differentiation deficiency^28,29^. Early genetic perturbation of CHD8 and other ASD risk genes in the developing mouse brain and human neural organoids indicated a regulatory role of the Wnt pathway^30–32^. Chromatin accessibility analyses in mouse stem cells have shown that CHD8 can bind and function in both accessible and highly inaccessible chromatin regions^33,34^. These results suggest that CHD8 can regulate proximal and distal promoter regions with various accessibility profiles. Furthermore, CHD8 expression across developmental stages and multiple brain regions is largely uniform^17,30,35^. Numerous studies proposed that CHD8 regulates the expression of autism risk genes in neural and non-neural cells^26,48^ raising the question of how CHD8 specifically affects neurons and how its absence can cause a common neuropsychiatric disorder.

Given the limited understanding of CHD8’s molecular functions in human neurons, we here generated a genetic loss-of-function model in human pluripotent stem cells and investigated the role of CHD8 in defined, functional stem cell-derived neurons^36^.

## Results

### Generation of a conditional loss-of-function allele of CHD8 in human excitatory neurons

**Figure 1:**
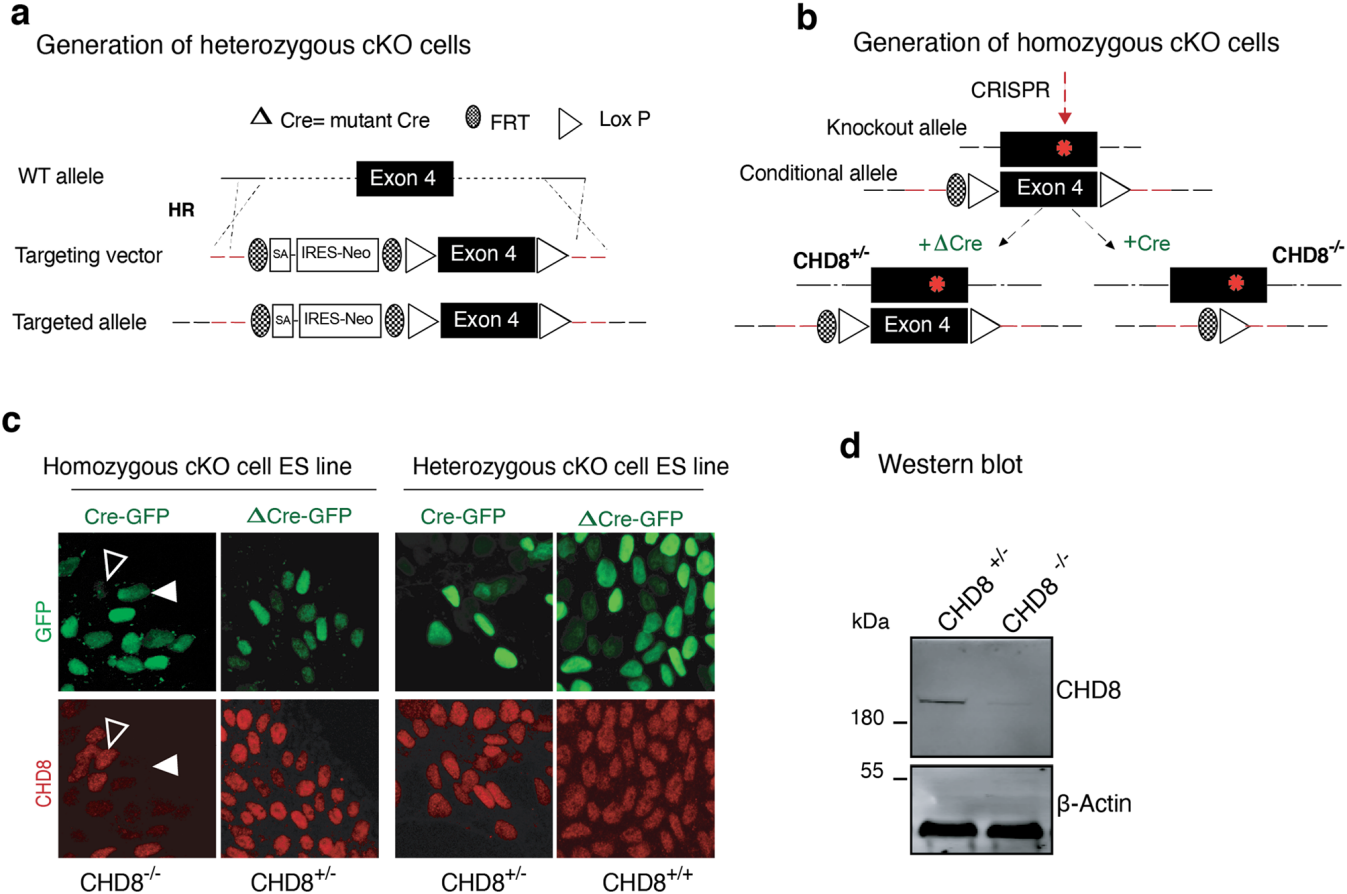
Generation of conditional CHD8 knockout stem cells. (**a**) Strategy to generate a heterozygous conditional knockout (cKO) allele of *CHD8* in pluripotent stem cells. The endogenous Exon 4 was flanked with LoxP sites by AAV-mediated homologous recombination. Following correct targeting, the selection cassette was removed by transient transfection with FlpE recombinase to generate the final conditional allele. Infection with Cre recombinase leads to deletion of the floxed allele and generates CHD8 KO cells. Infection with ΔCre (an inactive form of Cre) is used throughout the study for the control condition. (**b**) Generation of homozygous *CHD8* cKO cells by introducing a CRISPR transfection-mediated indel mutation into the non-conditional *CHD8* allele, which led to a frameshift mutation. Infection of a correctly targeted line with Cre recombinase generates homozygous CHD8 null cells, whereas control infection with ΔCre leaves the engineered mutations unchanged (see also Figs. 2a–d and 3a–d) (**c**) Left shows immunofluorescent images of CHD8 staining in conditional heterozygous and homozygous *CHD8* KO embryonic stem cells three days after infection with Cre/Δ Cre to detect CHD8 reduction. Right depicts the bright-field images of homozygous *CHD8* KO and control neurons, 23 days after in vitro differentiation assay. (**d**) Western blotting of heterozygous, and homozygous neurons (please see also the Fig. 3d).

**Figure 2:**
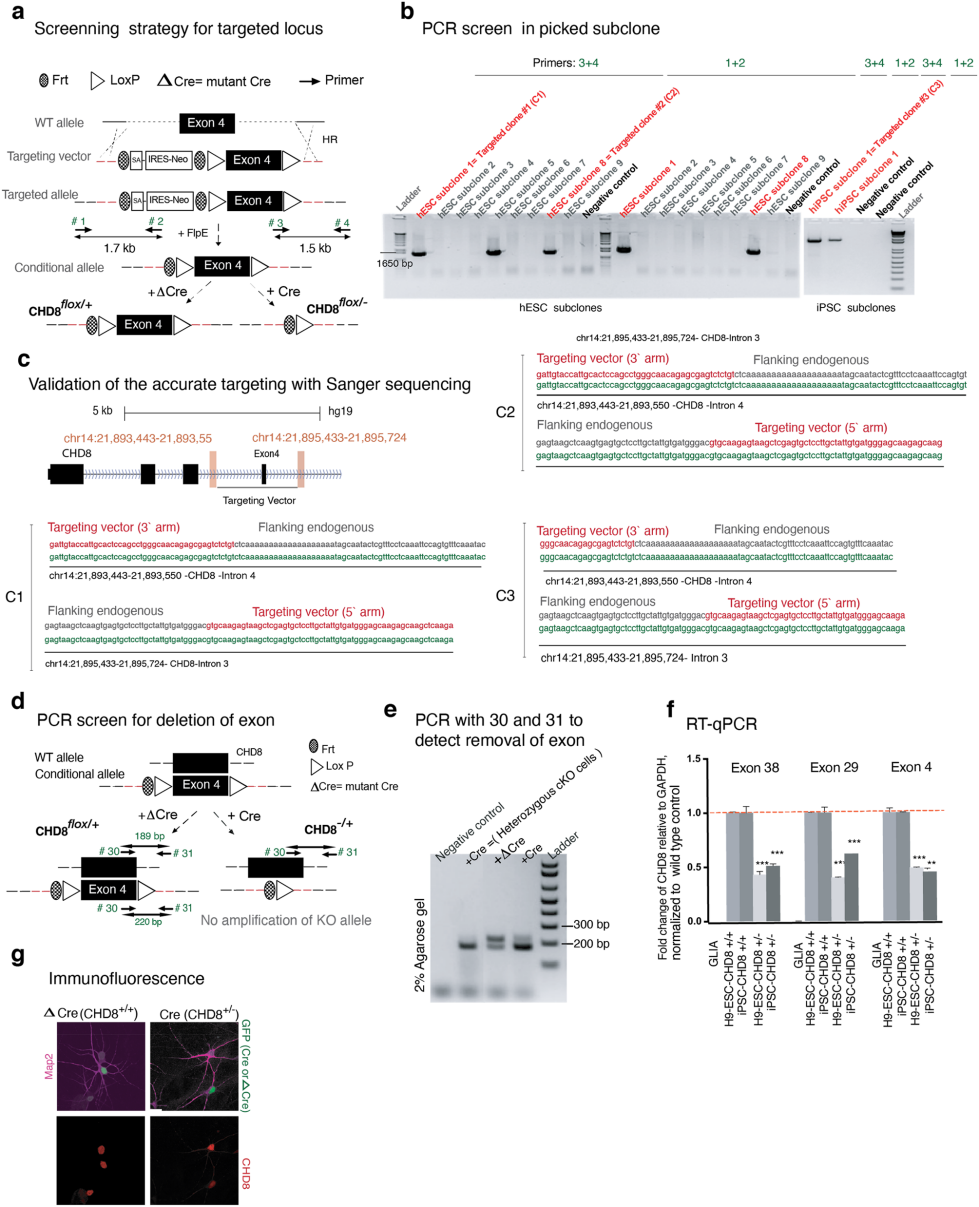
Targeting and characterizing conditional heterozygous CHD8 knockout stem cell lines. (**a**) Schematics of Targeting strategy for generating a conditional KO allele of the *CHD8* gene by flanking exon 4 with LoxP sites (similar to Fig. 1a with the inclusion of the ‘screening’ primers). Deletion of exon four is predicted to create a frameshift mutation with early truncation. (**b**) Screening PCR using external primers designed for outside the homology arm towards inside the targeting vector identified two subclones from the hESC line (C1 & C2) and one subclone from the iPSC line (C3) that were positive for the insertion of the targeting vector. (**c**) Sanger sequencing is spanning the targeting arms' transition into endogenous sequences, demonstrating correct targeting of the construct into the *CHD8* locus (clones C1, C2, and C3). (**d**, **e**) Excision of exon four after infection with LV-Cre and screening with the primers around the loxP sites (primer 30 and primer 31) resulted in a single band from heterozygous KO compared to two bands in WT cells as expected. (**f**) Quantitative reverse transcription PCR (RT-qPCR) using the probes for three exons of CHD8 gene shows the levels of mRNA decreases in heterozygous KO neurons. (**g**) Immunofluorescence analysis of heterozygous KO and WT neurons for Map2 and CHD8. The nuclear staining signal intensity significantly decreases in heterozygous mutant neurons.

**Figure 3:**
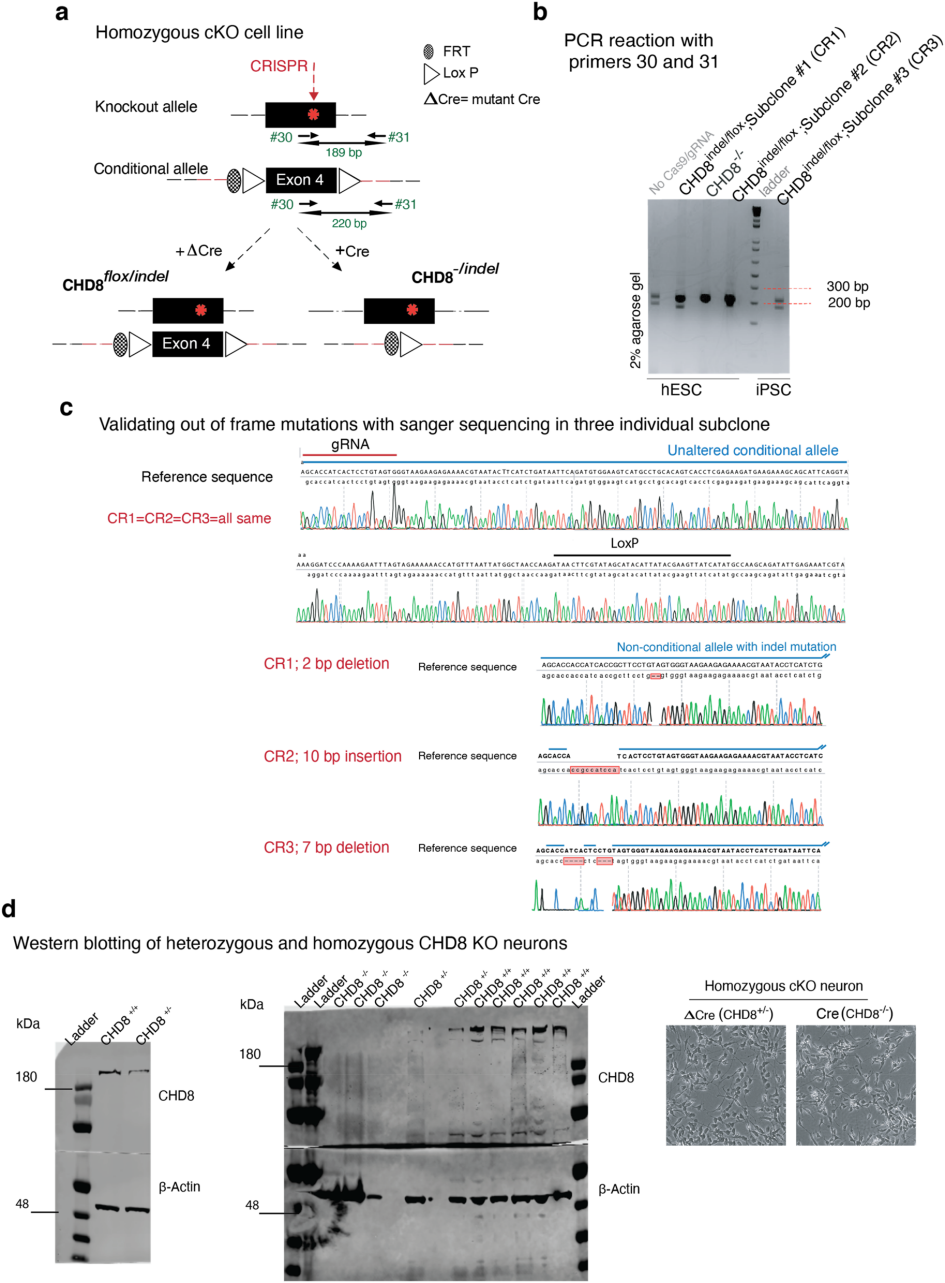
Targeting conditional homozygous CHD8 knockout stem cell lines. (**a**) Introduction of an indel mutation by CRISPR-CAS9 to non-conditional exon 4 of *CHD8* gene, to generate a conditional homozygous knock out cells. (**b**) Validation of the genotype by PCR around the loxP sequence (spanning the gRNA targeting region) and amplification of two bands; one allele is 32 bp smaller than the other allele. Therefore, the top band corresponds to the floxed allele, and the bottom band is the non-conditional allele, a candidate for carrying an indel mutation. Each band is cut and gel-purified, TOPO cloned, and sequenced using M13 forward and M13 reverse primers (CR1, CR2; both hESC and CR3, is an iPSC subclone, confirmed to carry an indel mutation in non-floxed allele). (**c**) Sanger sequencing of floxed and non-floxed alleles identified three subclones that carry frameshift indel mutations in the non-conditional allele with an un-altered floxed allele. Note that the conditional exon is shown only once as the representative sequence for all three subclones. (**d**) Western blotting of heterozygous and homozygous CHD8 knockout neurons reveals a decrease in total protein compared to the control condition (shows two independent replicates). The Right is the phase-contrast images of day 5 neurons and shows cultures of KO and control cells are indistinguishable.

To study the role of CHD8 in human neurons, we engineered the *CHD8* locus in pluripotent stem cells to produce heterozygous and homozygous conditional knockout (cKO) cells. The heterozygous cKO allele was constructed by surrounding exon four with two loxP sites (Figs. 1a and 2a). Deletion of exon four is predicted to produce a frameshift and early termination mutation. We produced two correctly targeted embryonic stem (ES) cell clones (C1&2) and one correctly targeted induced pluripotent (iPS) cell line (C3, see also Fig. 2b,c and characterization of the subclones in Fig. 2d–f). To generate a homozygous cKO of *CHD8*, we used CRISPR/Cas9 to introduce an indel mutation in the non-targeted wild-type allele of each of the three heterozygous cKO clones resulting in clones CR1-3 (Figs. 1b and 3a,b). The CR1 clone had a 2 bp deletion, CR2 had a ten bp insertion, and CR3 had a 7 bp deletion at non-conditional allele (Fig. 3c). Immunofluorescence and western blot analysis of neurons differentiated from human ESC clones revealed protein depletion in heterozygous and homozygous cKO cells (Figs. 1c, d and 2g, the full-length blots of technical replicates provided in Fig. 3d-left). Unlike in mouse embryos, lack of CHD8 in human neurons did not impact cell viability, enabling us to characterize the loss of function in differentiated, functional human neurons (Fig. 3d-right)^28^.

### CHD8 knockout neurons exhibit intact basic neuronal and synaptic function

**Figure 4:**
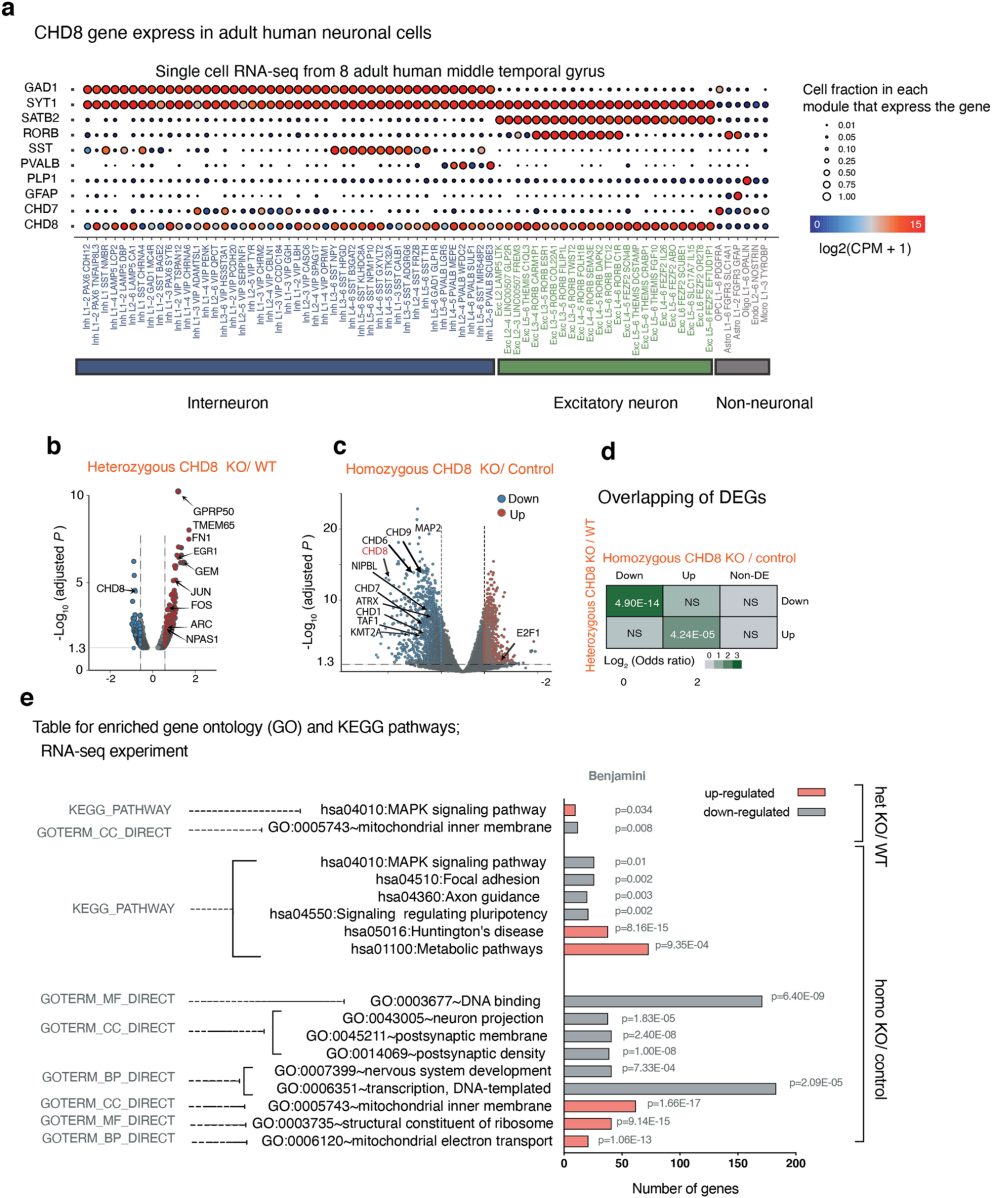
Loss of CHD8 leads to prominent gene downregulation in human excitatory neurons. (**a**) Single-cell RNA sequencing data showing expression of CHD8 and cell-type-specific marker genes (CPM + 1) in human cortical neurons (image credit: Allen Institute)^37^. (**b**) Volcano plot for RNA-seq fold change in heterozygous CHD8 KO versus control neurons. (**c**) Volcano plot for RNA-seq fold change in homozygous CHD8 KO versus control neurons. (**d**) Analysis for overlapping DEGs between the heterozygous and homozygous knockout RNA-seq experiment shows that the odds ratio of downregulated genes is significantly higher compared to overlapping upregulated genes. (**e**) Results of gene ontology (GO) and KEGG pathway analysis for up and downregulated genes in heterozygous *CHD8* KO to WT and homozygous *CHD8* KO to control conditions.

**Figure 5:**
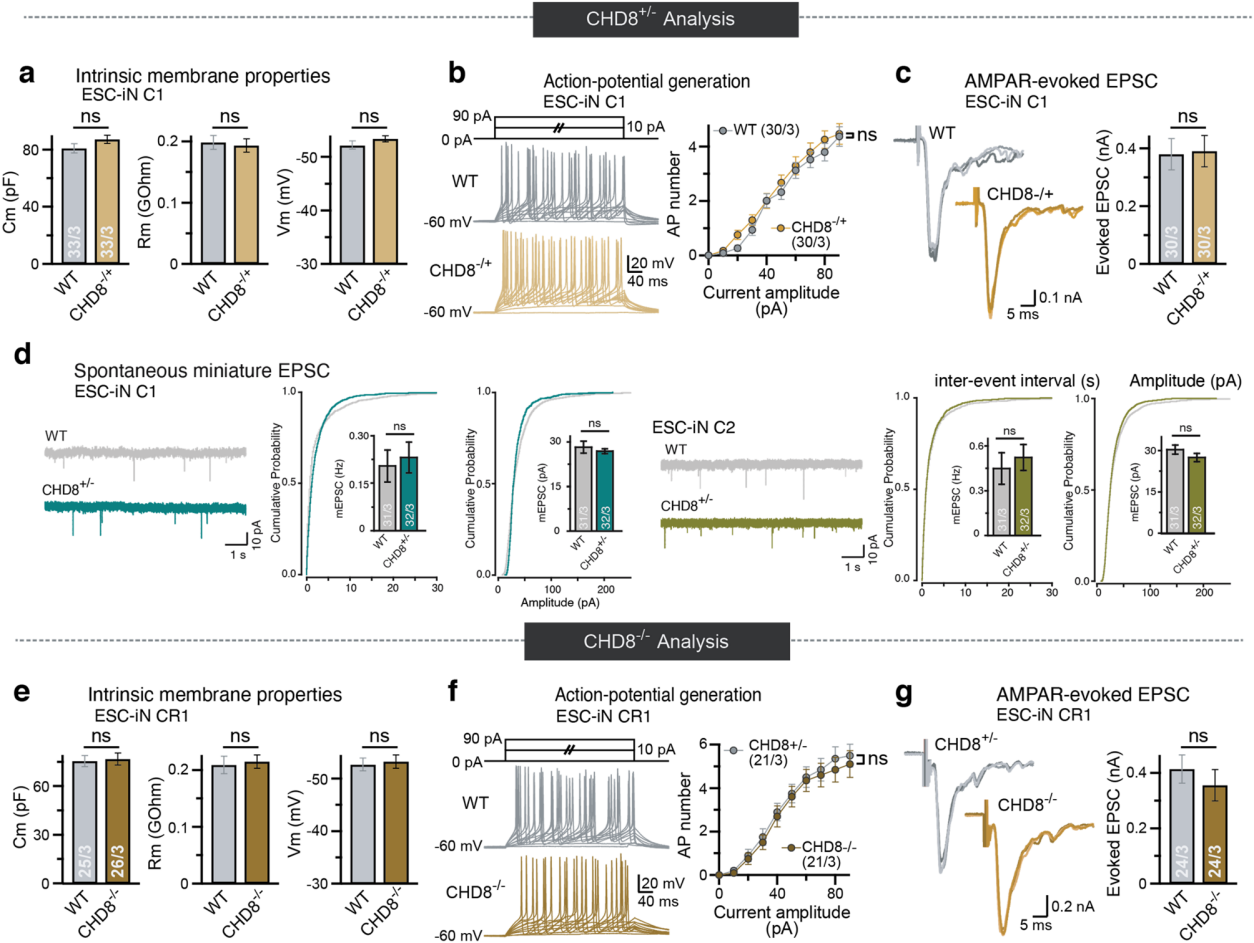
Electrophysiological characterization of heterozygous and homozygous conditional human excitatory neurons. (**a**) Electrophysiological recordings of the intrinsic membrane properties in the C1 neurons, a heterozygous conditional KO line: capacitance (Cm), resting membrane potential (Vm), and input resistance (Rm). N = 33 recorded cells in 3 batches (see numbers in bars). Student’s t-test. (**b**) Active membrane properties of C1 neurons demonstrated by stepwise current injection protocol. The number of action potentials in response to current amplitude is plotted (right). (**c**) Amplitude of evoked excitatory postsynaptic currents (EPSCs) in clone C1 showed no changes between the heterozygous KO and the WT neurons. (**d**) Amplitudes, and frequency of spontaneous miniature EPSC (mEPSCs) in the presence of 1 µM tetrodotoxin showed no change between *CHD8* heterozygous KO and WT neurons in clone C1 and clone C2, N = 31 or 32, respectively in 3 batches. Student’s t-test. (**e**) Analysis of the conditional homozygous mutant cell line CR1 neurons. Shown are capacitance, input resistance, and resting membrane potential. (**f**) Active membrane properties of CR1-derived neurons as in b. (**g**) Recording of evoked excitatory postsynaptic currents (EPSCs) CR1 derived neurons shows no statistically significant difference between Cre and ΔCre (*CHD8*^*−/−*^ vs. *CHD8*^+*/−*^) neurons. N = 24 cells in 3 batches, Student’s t-test.

Since single-cell RNA sequencing data from the middle temporal gyrus of the human adult cortex showed that *CHD8* is predominantly expressed in excitatory neurons, we sought to analyze the conditional CHD8 mutations in differentiated excitatory neurons from human pluripotent stem cells (Fig. 4a)^36,37^. To that end, we differentiated conditional cells to excitatory neurons and infected the cells with lentiviral vectors encoding Cre recombinase or ΔCre (inactive enzyme). Electrophysiological characterization of differentiated neurons revealed that intrinsic and active membrane properties of heterozygote and homozygote CHD8-mutant neurons were robust but not altered compared to control neurons (Fig. 5a,b,e,f). Additionally, mutant neurons formed functional synapses and the frequency and amplitude of spontaneous miniature EPSCs in CHD8 heterozygous cKO cells were not statistically different from control neurons (Fig. 5d). We further found that evoked excitatory postsynaptic currents (EPSCs) were unchanged in heterozygous and homozygous mutant cells (Fig. 5c,g). Thus, loss of *CHD8* did not affect the gross intrinsic physiological and synaptic properties using standard electrophysiology in cultured human neurons suggesting that more subtle functional phenotypes may lead to neuronal dysfunction in CHD8-mutant patients’ brains.

### CHD8 regulates actively transcribed genes

Next, we evaluated the transcriptional effects of CHD8 depletion in neurons. RNA sequencing showed that heterozygous *CHD8* mutant cells exhibit only subtle transcriptional changes compared to WT cells, confirming previous reports (Fig. 4b)^26,38^. Homozygous knockout neurons, on the other hand, showed pronounced transcriptional changes, with substantially more downregulated genes than upregulated genes (Fig. 4c). The transcriptional change in genes which were downregulated in both heterozygous and homozygous KO cells was more pronounced than that observed in overlapping upregulated genes (Fig. 4d). These results suggest that CHD8 is a transcriptional activator in human neurons.

**Figure 6:**
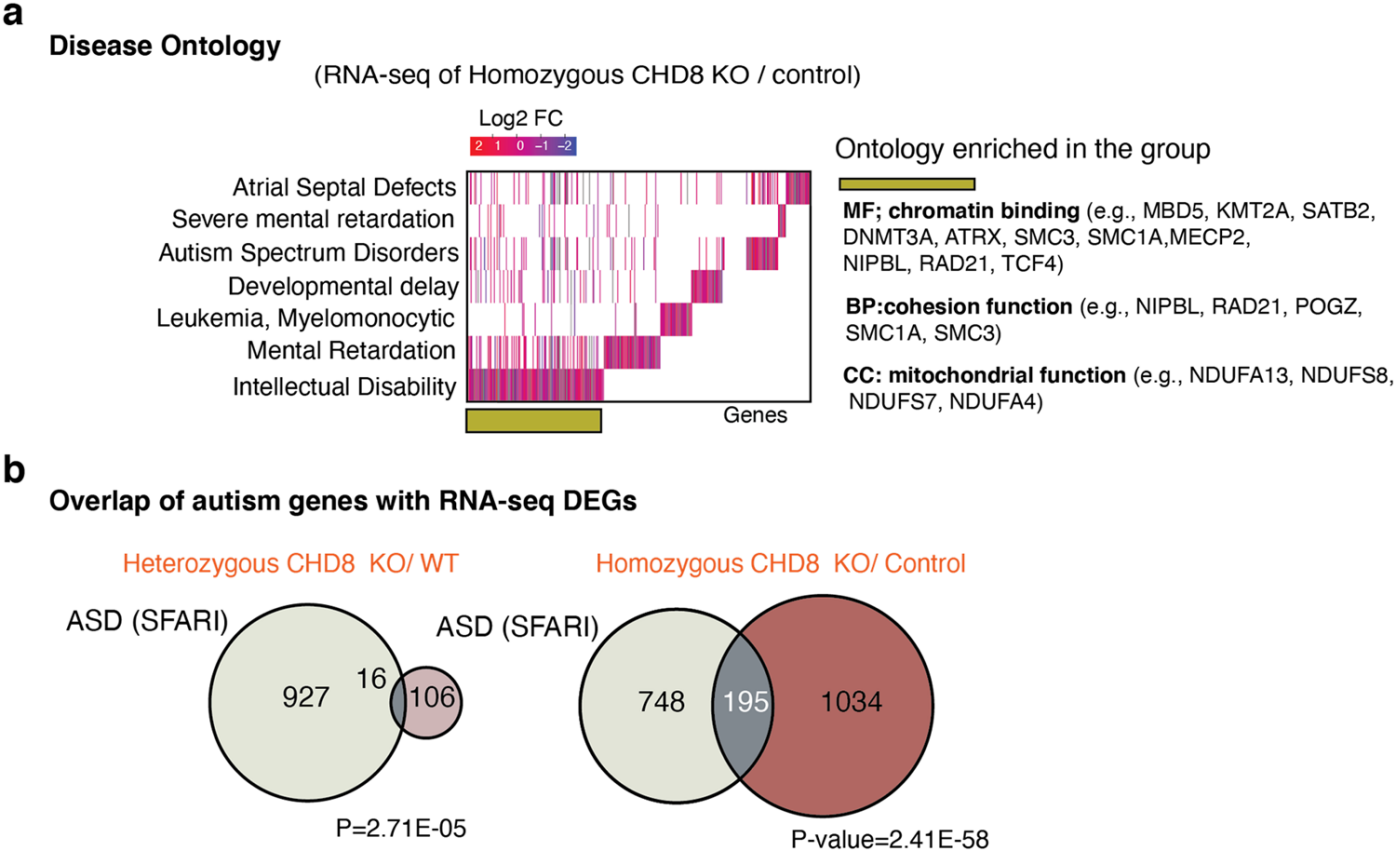
Genes de-regulated in CHD8 KO neurons are enrichted in genes related to neurodevelopmental disorders among other diseases. (**a**) Disease Ontology (DO) and enrichment analysis for DEGs from the homozygous RNA-seq experiment shows enrichment of genes for human diseases (left). Additionally, we annotated molecular and biological ontology within the group of genes involved in the pathology of intellectual disability (right)^39^ (see also Fig. 7a). (**b**) Venn diagrams depict the number of overlapping ASD genes (from SFARI database) and CHD8 target DE gene from results obtained in RNA-Seq experiments.

**Figure 7:**
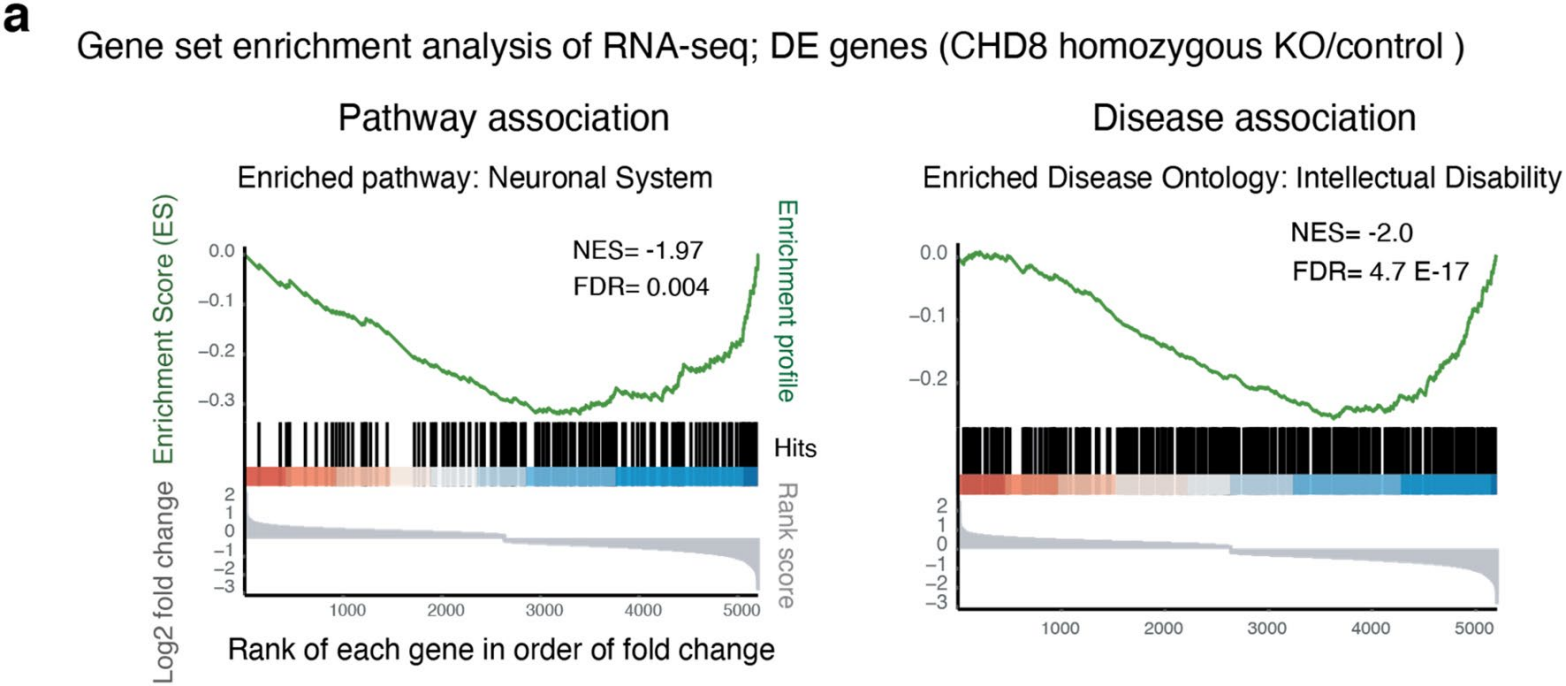
Analysis of gene expression changes in CHD8 KO neurons a, Gene Set Enrichment Analysis (GSEA) of the pathways and diseases for DE genes from *CHD8* homozygous KO neurons.

Gene ontology (GO) and pathway enrichment analysis of heterozygous and homozygous CHD8 reveals CHD8 regulates ASD genes knockout cell transcriptomes demonstrated that CHD8 uniformly affects genes involved in neuronal function (axon and synapse development) and signaling pathways related to MAPK/ERK signaling (Fig. 4e). Furthermore, disease pathway enrichment and disease-associated Gene Set Enrichment Analysis (GSEA) of DE genes reveal that CHD8 depletion influences gene signatures commonly associated with neurodevelopmental disease (Figs. 6a and 7a-left). Given its genetic association with autism we interrogated the expression changes of established autism-risk genes and indeed found an overrepresentation of these genes among all CHD8-regulated genes (Figs. 6b and 7a-right)^46^.

### CHD8 binds at the promoters of actively transcribed genes in neurons

**Figure 8:**
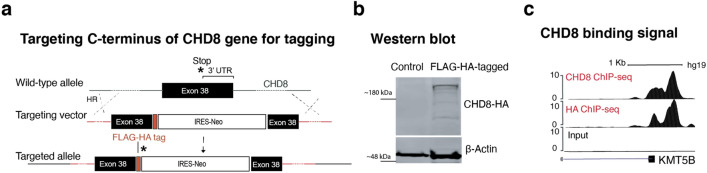
ChIP-seq of endogenously tagged CHD8 in stem cells revealed binding to its previously described target promoters. (**a**) Targeting strategy to insert a C-terminal FLAG-HA tag at the endogenous *CHD8* locus (see Fig. 3a–d). (**b**) Western blot analysis of tagged and non-tagged (control) ES cells using an anti-HA antibody (see the full-length version of this blot in Fig. 9e). (**c**) A CHD8 peak at promoter of the *KMT5B* gene.

**Figure 9:**
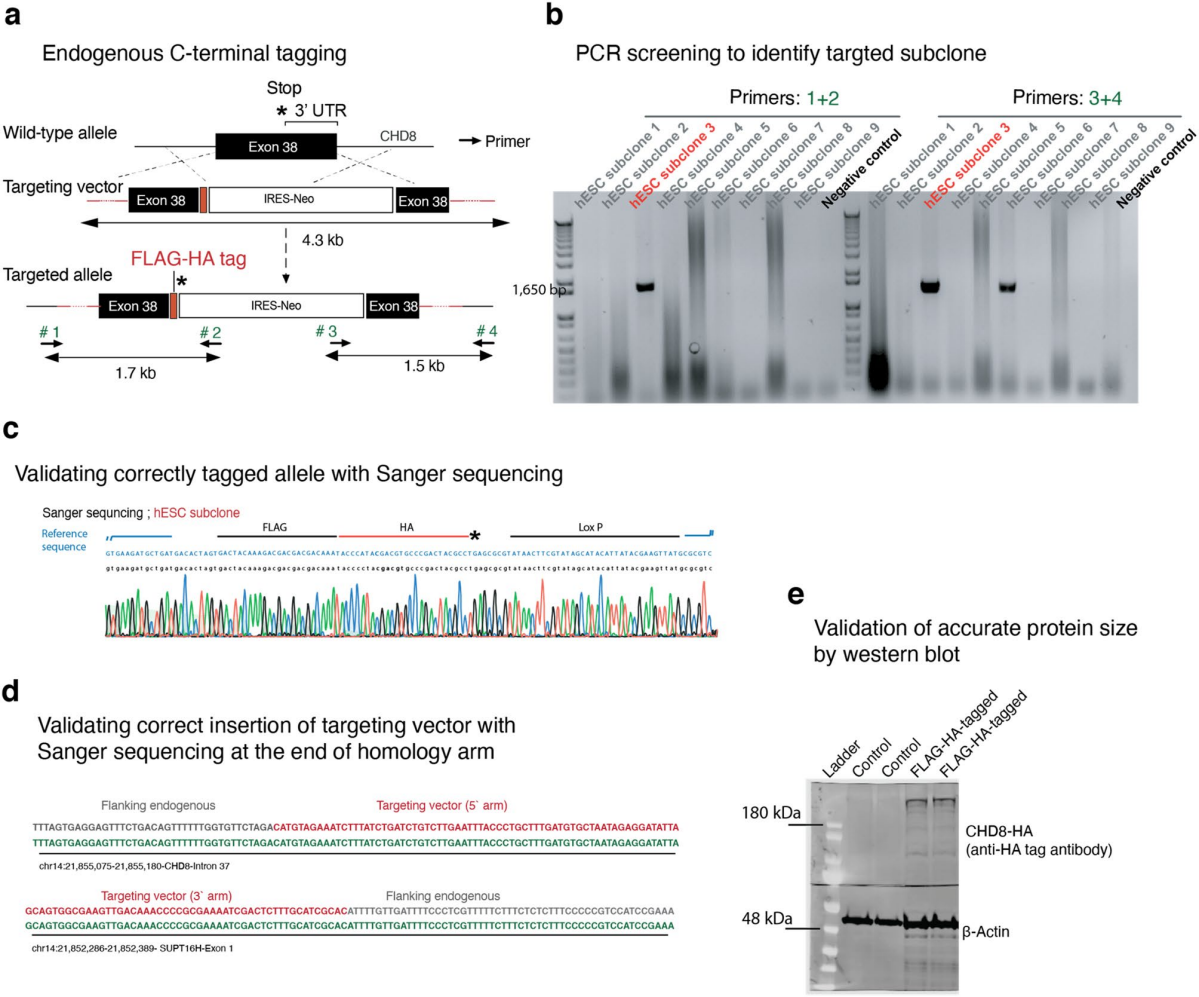
FLAG-HA tagging of endogenous CHD8 protein. (**a**) Schematic shows targeting strategy for in-frame insertion of FLAG-HA tag at the C-terminus region of CHD8 gene (similar to Fig. 11a with inclusion of the ‘screening’ primers). Throughout the manuscript, we have used the HA tag for downstream experiments. (**b**) Screening PCR from Neomycin resistant hESC colonies with external primers, i.e., one primer outside of the homology arms and one primer inside the targeting vector (primer #1 and #4). (**c**) Sanger sequencing to detect a correct insertion of donor vector in the C-terminus of CHD8 gene within its frame and before the STOP (*) codon. (**d**) Sanger sequencing to validate the correct transition of targeting vector into endogenous arms (black line after the blue lines on both sides). (**e**) Western blotting reveals correct protein size for HA-tagged CHD8 protein (The blot is indeed the full-length version of the same blot shown in Fig. 8b).

**Figure 10:**
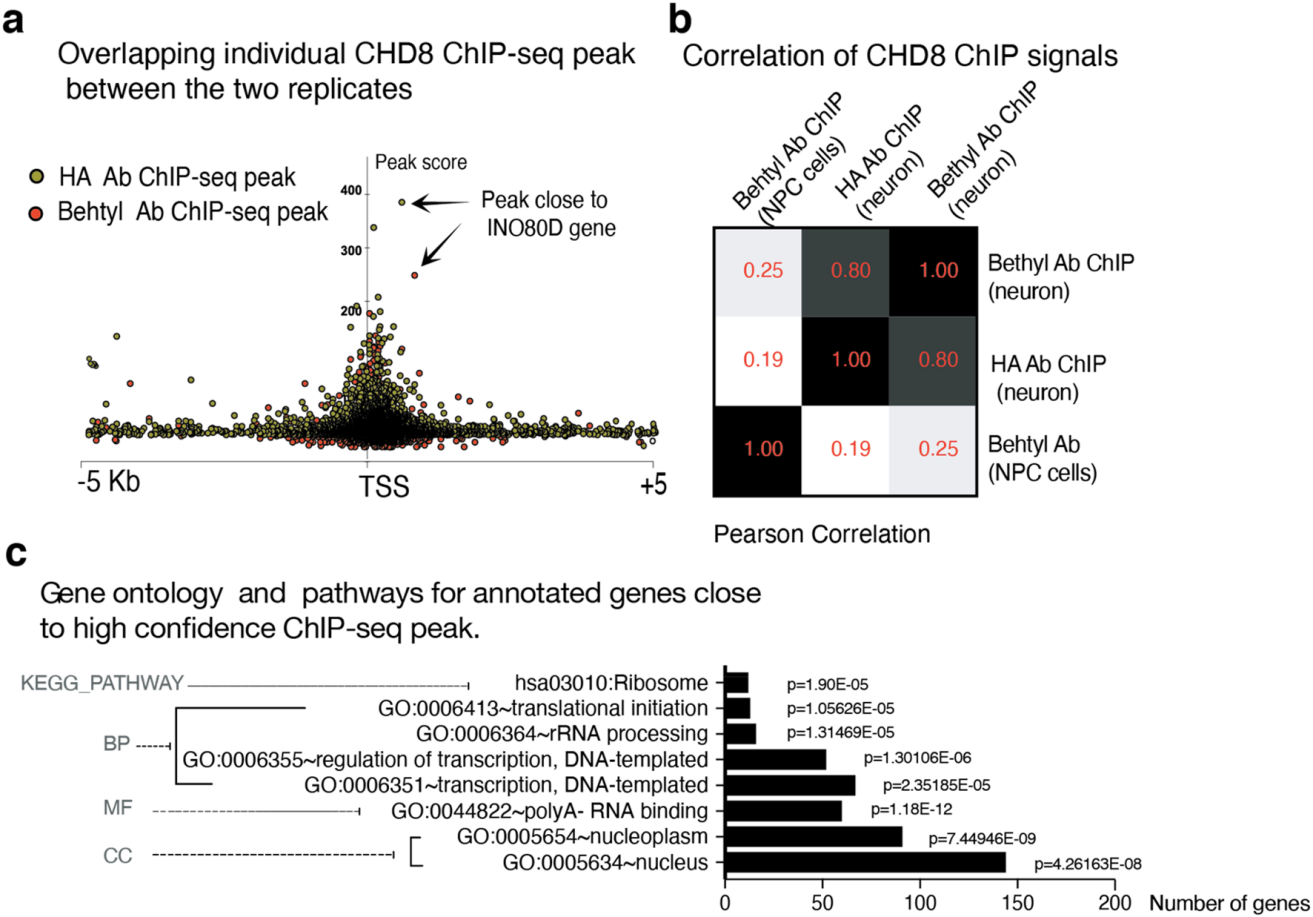
Characterization CHD8 ChIP-Seq binding. (**a**) Individual CHD8 ChIP-seq peaks from two replicates that were pulled down with two different antibodies (anti-CHD8 and Rabbit anti HA). (**b**) Pearson's correlation between our two CHD8 ChIP-seq signals in neurons and a previously published CHD8 ChIP-seq dataset in NPC cells on selected, overlapping peak sites (GSE61492)^40^. Signals are compared on a set of selected, overlapping peak sites (10,000 peaks). (**c**) Enriched signaling pathways and ontology among genes with CHD8 peak at their promoters (± 5 Kb distance).

**Figure 11:**
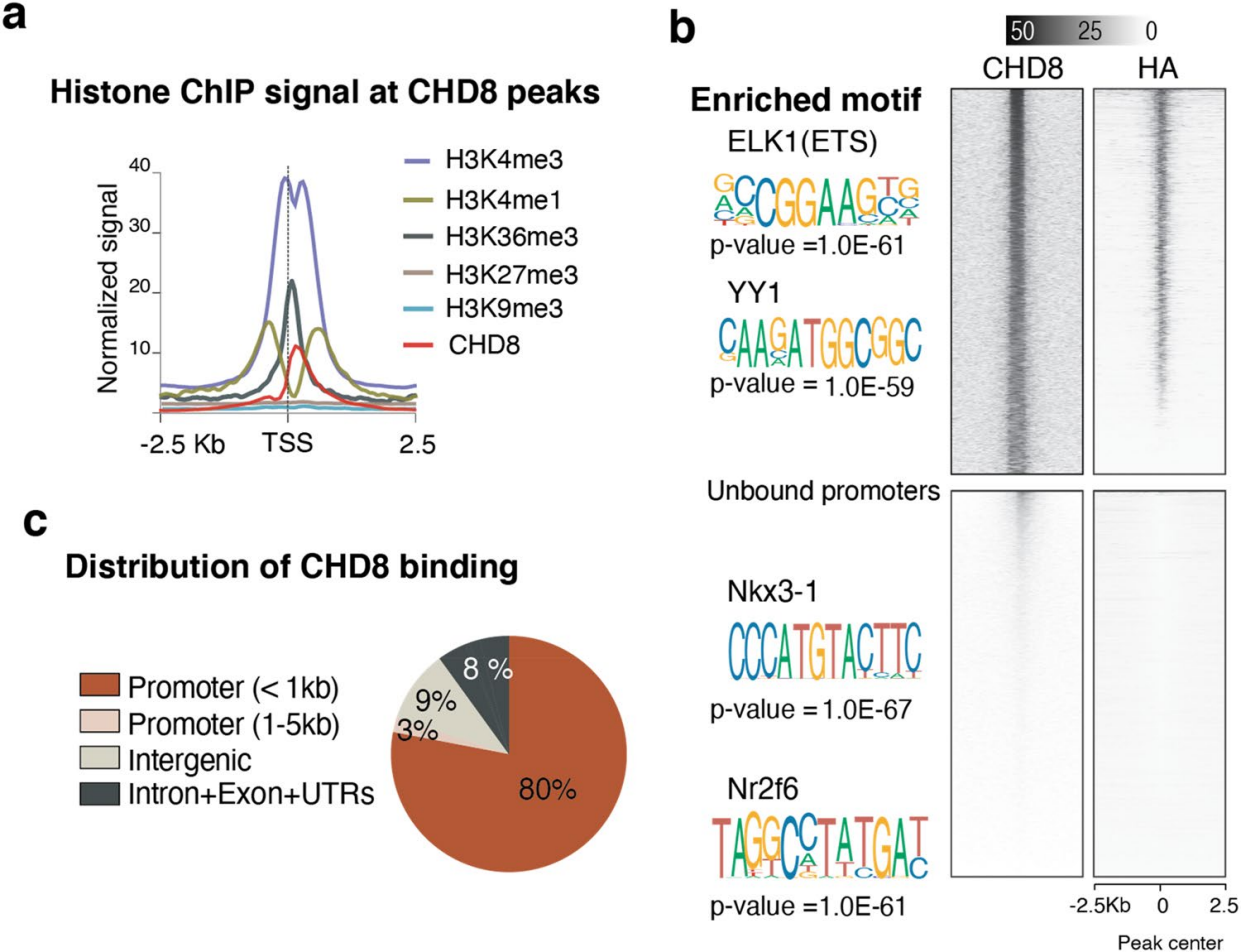
CHD8 binds to active promoters enriched with ELK1 motif. (**a**) CHD8 peaks overlapping with active histone modifications in neurons, (analysis of ENCODE data for H9 cell-derived neurons)^41^. (**b**) Top heatmaps are CHD8 binding at overlapping peaks between two separate pull-downs of HA and CHD8 antibodies (n = 3696 overlapping peaks). Using HOMER and MEME, we found ETS and YY1 motifs enriched at CHD8-bound sites. The bottom panels' heatmaps are signals from the same samples that stratified on CHD8-unbound promoters (n = 4000). The greyscale shows normalized coverage for all groups^42,43^. (**c**) Pie chart shows the distribution of CHD8 ChIP-seq peaks across the genomic regions in ES cell-derived neurons.

**Figure 12:**
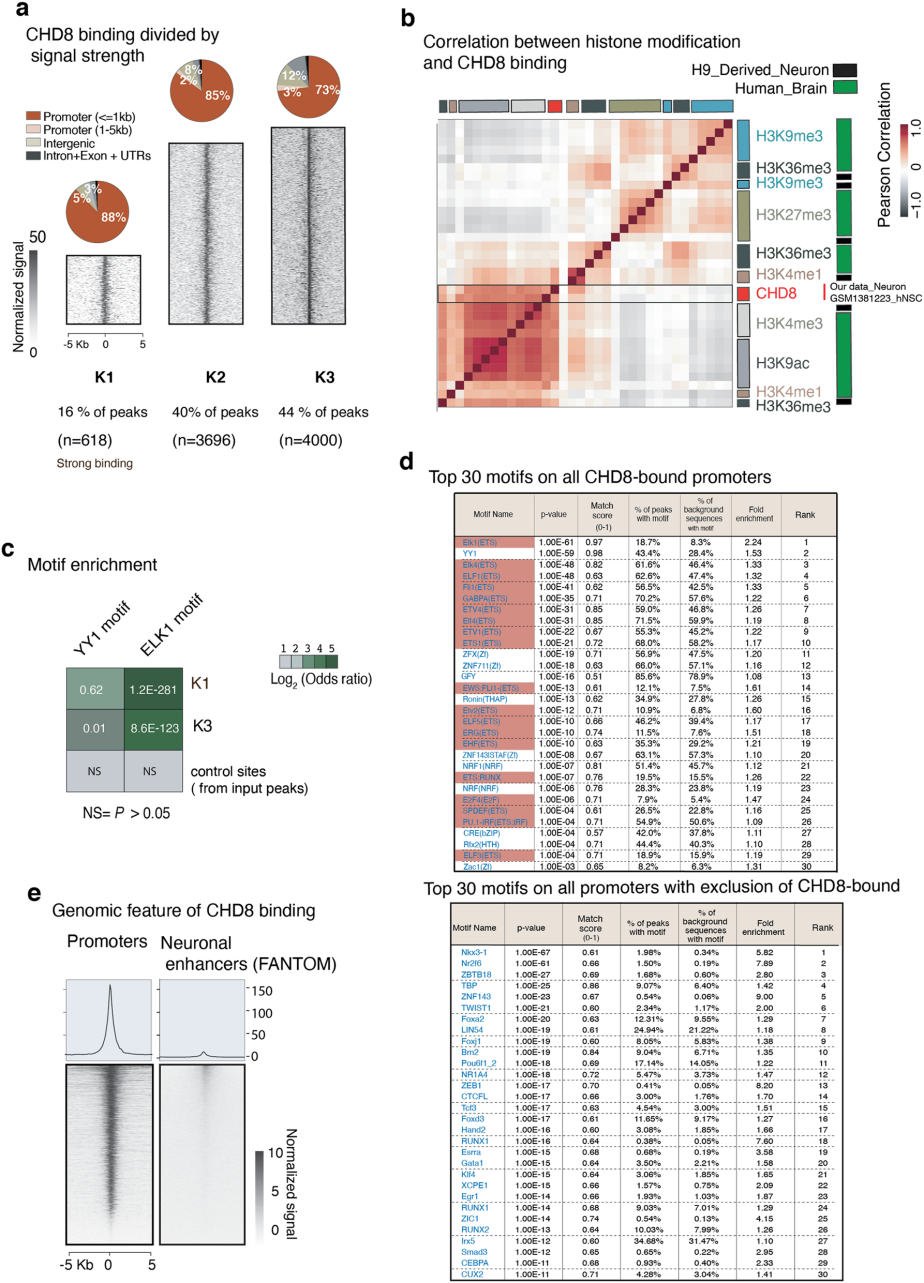
CHD8 binding and enrichment at chromatin regions with distinct histone modification in neurons. (**a**) K-means clustering ChIP-seq signal into three groups recognize the most specific targets of CHD8 binding at promoters. Genomic annotation and functional classification are separately assigned for each group. (**b**) Pearson`s correlation of histone modification obtained from H9 derived human neurons (ENCODE`s histone ChIP-seq data) and our CHD8 ChIP-seq signal at CHD8 binding sites (K1 + K2 + K3). (**c**) The odds ratio and the calculations of motif enrichment at strong CHD8 binding sites (K1), the weak binding sites (K3), and the control sites (randomly shuffled peaks from input). Sites (K1, K2, and K3) taken from clustering analysis of peaks among CHD8 binding sites-the top heatmap Fig. 13b). (**d**) Top 30 motifs enriched at the prompters (± 5 Kb) of 3000 CHD8-bound target genes. The ETS factor motifs are highlighted in red. (**e**) Heatmap of CHD8 binding signal at promoters of neural genes and enhancers (FANTOM database)^45^.

To map chromatin binding targets of CHD8 in human neurons, we generated a human embryonic stem (ES) cell line endogenously tagged at the C-terminus of the CHD8 gene with the FLAG-HA epitope sequence (Figs. 8a and 9a–d). Western blotting demonstrated that the tagged protein ran at the expected size of CHD8 (Fig. 8b, the full-length blot is provided in Fig. 9e). Next, we performed chromatin immunoprecipitation followed by sequencing (ChIP-seq) on differentiated human ESC-derived neurons using antibodies for both the HA-tag and the N-terminus of the CHD8 protein. We detected enrichment of CHD8 binding on promoters of known CHD8 target genes, such as lysine methyltransferase (KMT5B)^26^ (Fig. 8c). The binding profiles of CHD8 pulled down by the two antibodies were highly correlated (Pearson *r*^2^ = 0.80, Fig. 10a,b). Weak correlation of ChIP-seq signal with published ChIP-seq dataset from neural progenitors suggests that CHD8 binding is cell context-dependent (Pearson *r*^*2*^ = 0.25)^40^. We found a pronounced enrichment of promoter sequences among CHD8 binding sites (80% of CHD8 peaks) and a survey of various histone marks from the ENCODE repository revealed enrichment of active histone marks at neuronal promoters overlapping CHD8 peaks (Figs. 11a,c and 12a,b).Ontology terms of genes significantly associated with CHD8-bound promoters are related to chromatin biology, transcription, and translation (Fig. 10c)^41^. These observations suggest that the genomic binding of CHD8 is generally related to pathways governing gene regulation.

### The ETS motif is enriched among CHD8 binding sites

Motif enrichment analysis showed that CHD8 binding in human neurons is enriched for the ETS and YY1 motifs (Fig. 11b). The odds ratio of ETS motifs enrichment was significantly higher in the strong binding sites of CHD8 than weaker binding sites (Fig. 12c)^44^. Indeed, motif discovery analysis across multiple databases revealed that the top 30 enriched motifs within CHD8 binding sites were specific to ETS factors (Fig. 12d, ETS motifs highlighted in red). Classification of strong CHD8 peaks in neurons revealed a distinct enrichment around the proximal promoters and little binding at distal regulatory and enhancer sites (Fig. 12e)^45^.

### CHD8 acts as transcriptional activator in human neurons

**Figure 13:**
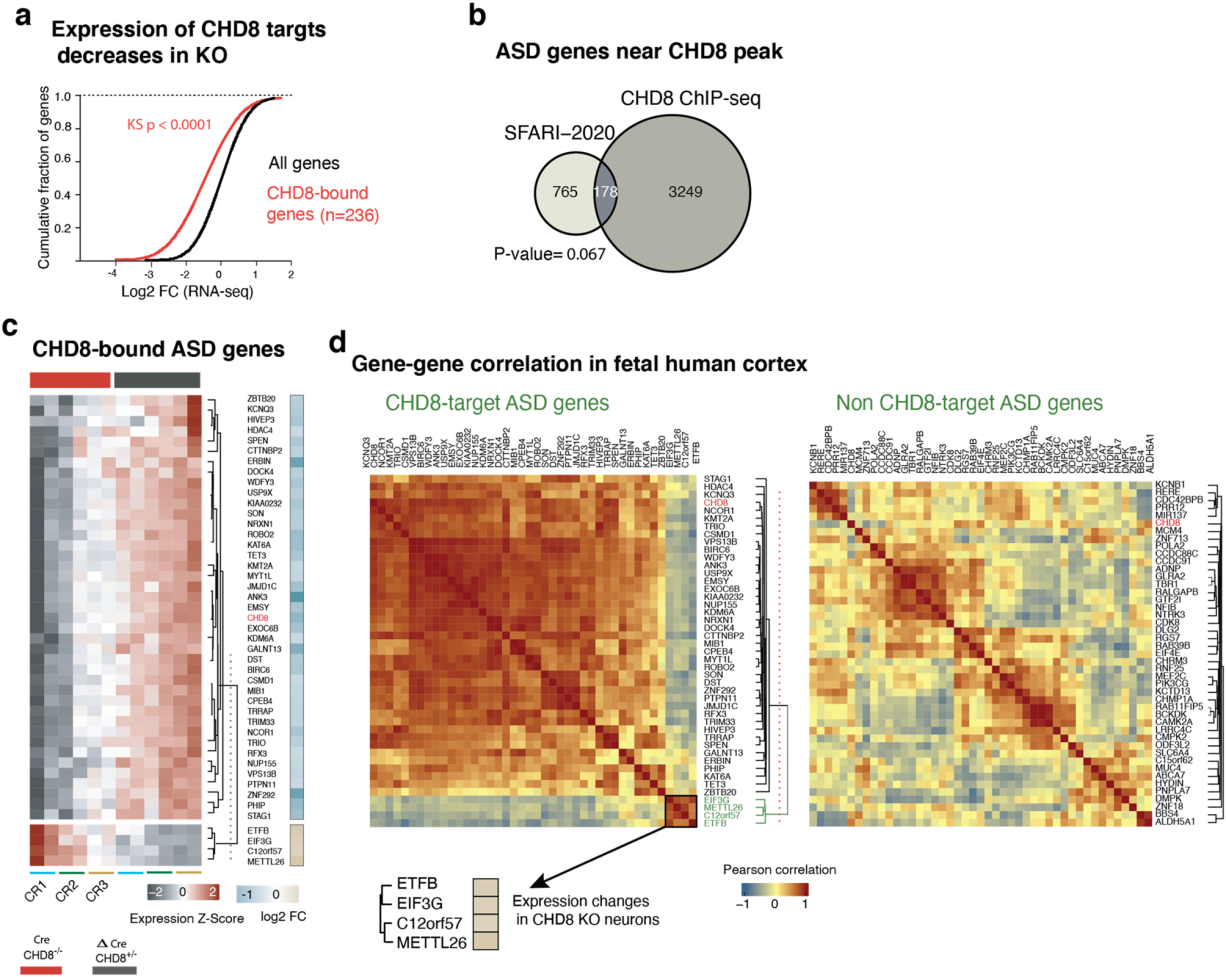
CHD8 positively regulates transcription of its target promoters and the ASD gene module that co-expresses in human brain. (**a**) Expression of genes with CHD8 peak at the ± 5 Kb of the promoters showed a marked decrease compared to control. (**b**) Overlapping of ASD genes from SFARI-2020 list and genes with CHD8 binding at promoters^46,47^. Significance of the overlapping calculated with the hypergeometric test. (**c**) Gene expression of CHD8-bound ASD genes with significant change in RNA-seq experiment. (**d**) correlation analysis of CHD8-target ASD genes (same gene set we discovered in “**c**”) within the human fetal cortex (12–37 CPW) is plotted. The clusters of similarly expressed genes led to the separation of the same gene modules shown in “(**c**)”. A separate module of non-CHD8 target autism gene was randomly selected for correlation analysis of the control group.

**Figure 14:**
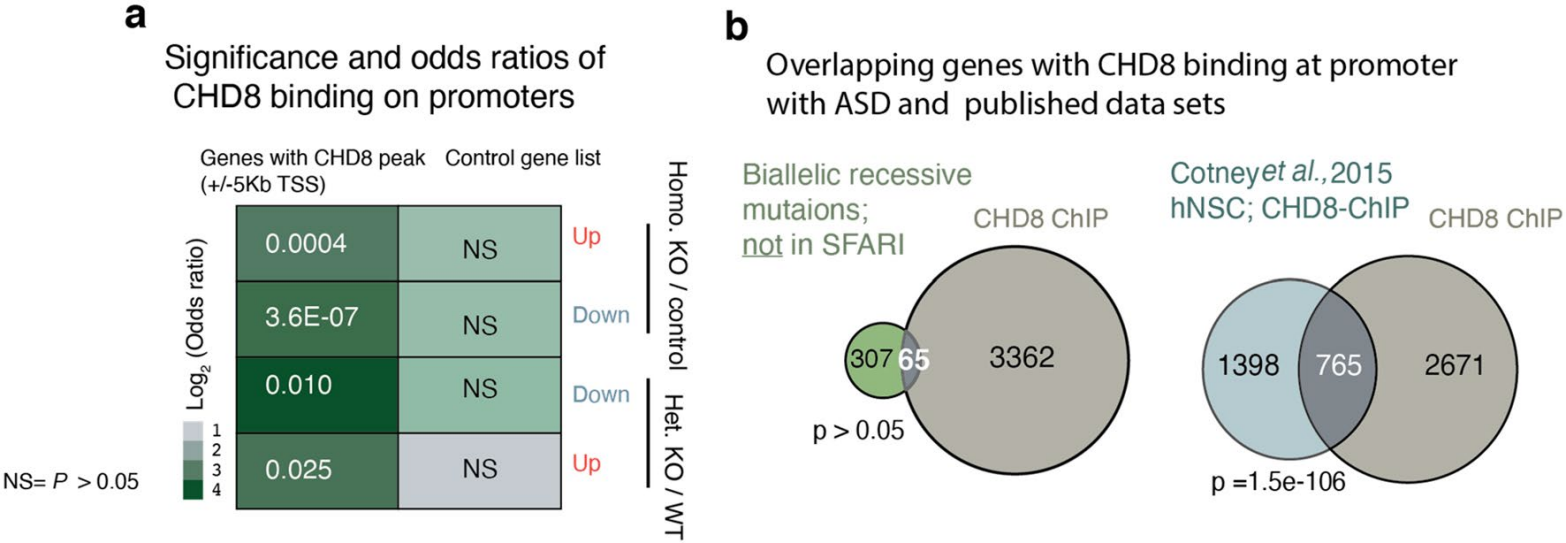
CHD8 binding at promoter of DE and ASD genes. (**a**) The odds ratio, strength, and significance of CHD8 binding on DEG promoters in heterozygous and homozygous CHD8 KO neurons (DE genes are obtained from RNA-seq experiment and odds ratio calculated based on overlapping gene list analysis) (GeneOverlap: http://shenlab-sinai.github.io/shenlab-sinai/). (**b**) Overlapping CHD8-bound genes in our experiment with genes that exhibit a recessive mutation in ASD and with previously published data set of CHD8 binding in human NPCs^48,49^. Significance of the overlapping calculated with the hypergeometric test.

Having mapped CHD8 binding in neurons, we next investigated the functional consequences on local chromatin and transcription at CHD8 target sites in response to CHD8 depletion. RNA-sequencing between control and CHD8 KO cells in the cumulative distribution of CHD8-bound and unbound genes showed that the loss of CHD8 leads to downregulation of its target genes, suggesting that CHD8 primarily acts as a transcriptional activator of its direct target genes (Fig. 13a). The observation that CHD8 binding is stronger at promoters of downregulated genes than of up-regulated genes and an overall gene downregulation in CHD8 KO neurons supports this conclusion (Fig. 14a). The expression analysis of overlapping autism genes revealed that CHD8 is required for their active transcription; therefore, majority of these genes are upregulated in CHD8 KO (Figs. 13b,c and 14b). Intriguingly, a subset of these autism genes that upregulated show distinct correlation of gene expression in the human fetal cortex (Fig. 13d; group of four genes in Fig. 13c also cluster in Fig. 13d)^37^.

### CHD8 promotes chromatin accessibility

**Figure 15:**
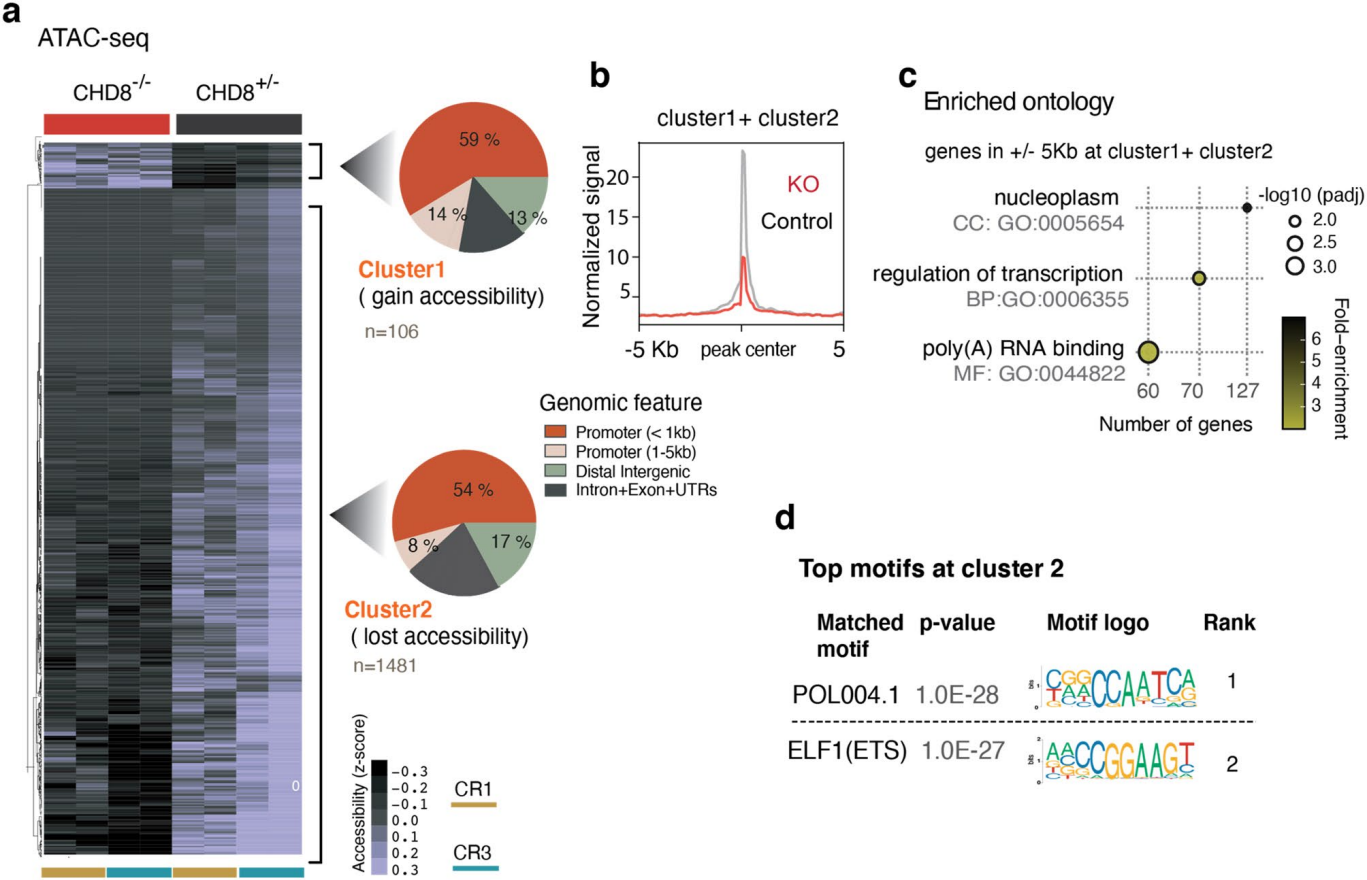
CHD8 globally promotes chromatin accessibility in neurons. (**a**) Heatmaps of normalized ATAC-seq signal in cKO homozygous and control neurons from a targeted ES cell line (CR1) and a targeted iPS cell line (CR3) and two technical replicates within each line. Cluster1 and Cluster 2 are separated based on unsupervised clustering analysis, which shows sites that gain or lost accessibility regions in KO and plotted the corresponding genomic annotation at each cluster. (**b**) Normalized ATAC-seq signal from aggregates of Cluster1 and Cluster2 shows KO chromatin predominantly lost accessibility. (**c**) Ontology analysis of the genes with differentially regulated ATAC-seq peaks in KO at the promoters' vicinity (± 5 Kb TSS in Cluster1 + Cluster2). (**d**) Motif enrichment analysis in Cluster 2.

**Figure 16:**
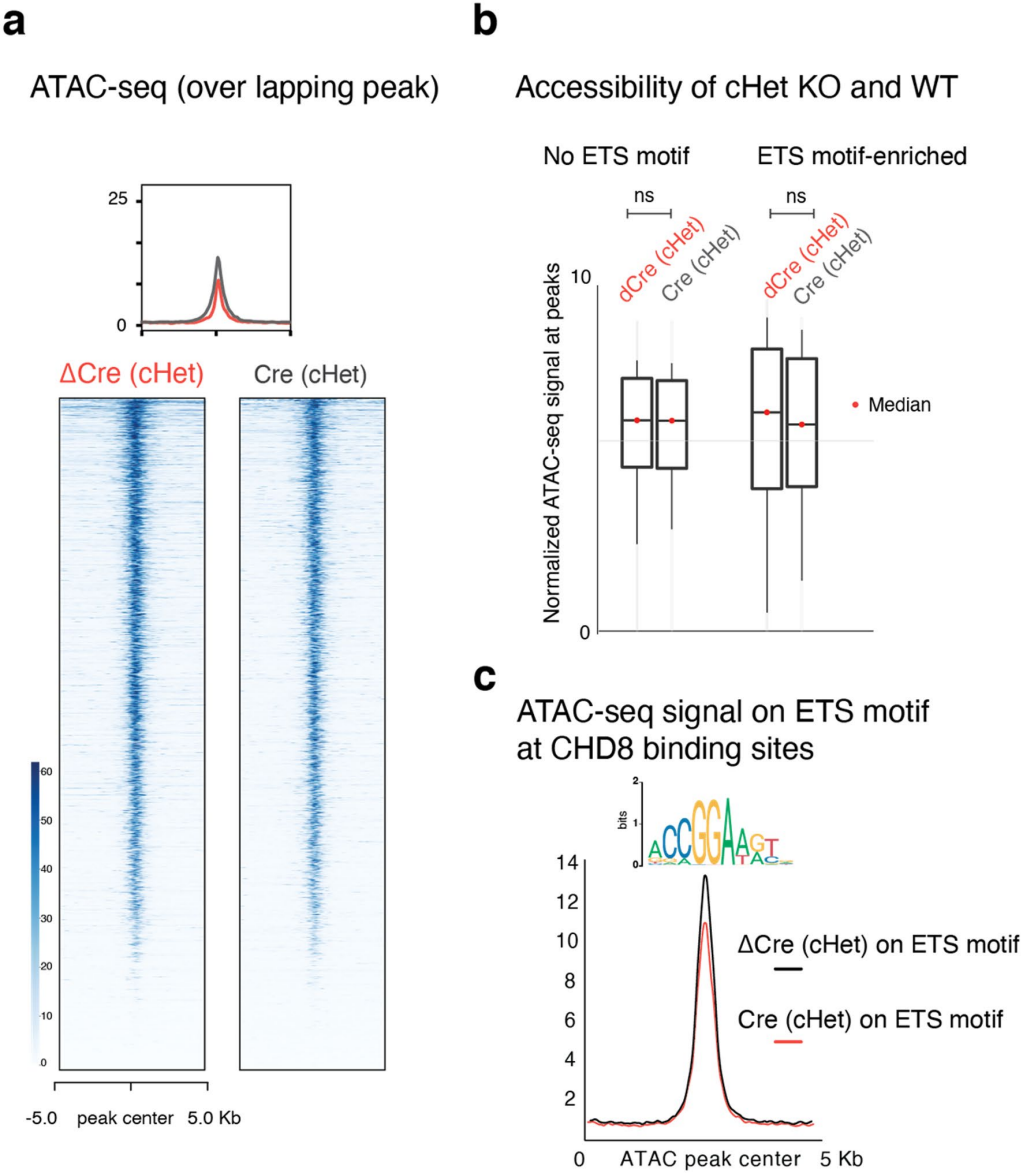
Comparison of ATAC-Seq results between heterozygous CHD8 knockout and WT neurons. (**a**) Heatmaps depicts normalized ATAC-seq signal across the overlapping peaks in wild type and heterozygous CHD8 knockout neurons. (**b**) Boxplots of chromatin accessibility at ETS motif factor sites in wild type and heterozygous CHD8 knockout neurons (ATAC peaks with more than one ETS motifs within each peak included in this analysis). The p-value is calculated with a non-parametric two-sided KS-test to compare the distribution of signals across the ATAC-seq peaks between the samples. (**c**) The enrichment plot of ATAC-seq signal from wild type and heterozygous CHD8 knockout neurons at selected sites that overlap with CHD8 binding in neurons and also carrying ETS factor motif**.**

Next, we characterized the chromatin remodeling activity of CHD8 in human neurons, utilizing Assay of Transposase Accessible Chromatin (ATAC)-seq^50,51^. Differential accessibility analysis of CHD8 heterozygous and homozygous knockout neurons revealed that many more sites lost accessibility (1,481 peaks in homozygous KO) than gained accessibility (106 peaks in homozygous KO) (Figs. 15a,b and 16a). These findings align with our previous results showing that CHD8 acts primarily as a transcriptional activator in neurons.

The ontology enrichment analysis for genes with a significantly changed ATAC-peak signal at their promoter vicinity (± 5 Kb) revealed transcriptional and RNA processing pathways (Fig. 15c). Motif enrichment showed significant overrepresentation of CAAT (an RNA polymerase II binding sequence) and the GGAA (the ETS factor motif) at sites that lost accessibility in CHD8 knockout neurons (Fig. 15d). The CAAT-box enrichment commonly found at core promoters, whereas the ETS motif is not specific to promoter regions^52^. Thus, this finding shows CHD8 play distinct role in activating ETS-containing sites, not in general promoter regions. The ATAC-seq peaks in the heterozygous knockout experiment at sites with moderate change (no statistical significance) also revealed similar results for ETS motif enrichment, suggesting mild but specific effect of CHD8 mutation on chromatin accessibility at ETS motif regions (Fig. 16b,c).

**Figure 17:**
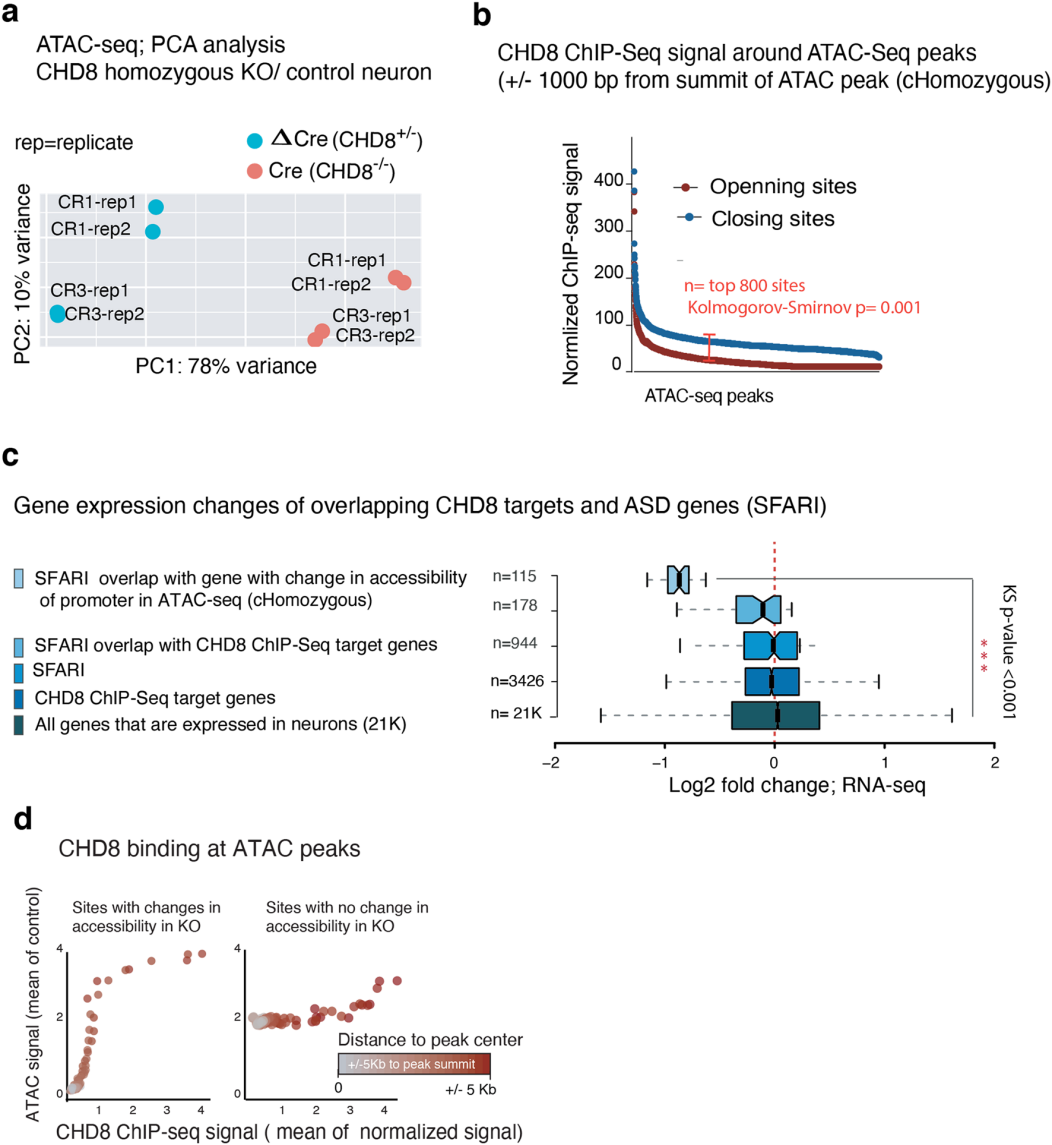
Analysis of ATAC-Seq and RNA-Seq from homozygous CHD8 knockout neurons. (**a**) Principal component analysis (PCA) for ATAC-seq signal from control (ΔCre) and homozygous KO (Cre) neurons and the separation of the samples based on the genotype in PC1. One embryonic stem cell line (CR1) and the One iPSC line (CR3), and two technical replicates for each line are used in the experiment. (**b**) CHD8 binding on sites of chromatin that exhibit accessibility change in ATAC-seq- experiment (homozygous KO) (CHD8 signal is taken from ± 1000 bp of the summit of ATAC-seq DE peak). (**c**) Boxplots represent log2 scaled fold change in RNA expression of CHD8 target genes including overlapping with autism gene list and control gene list (two-sample Kolmogorov–Smirnov test). (**d**) Analysis of CHD8 binding at *CHD8* KO ATAC-seq sites with differential accessibility and in regions with no change. The average ATAC-seq signal is calculated from control samples.

**Figure 18:**
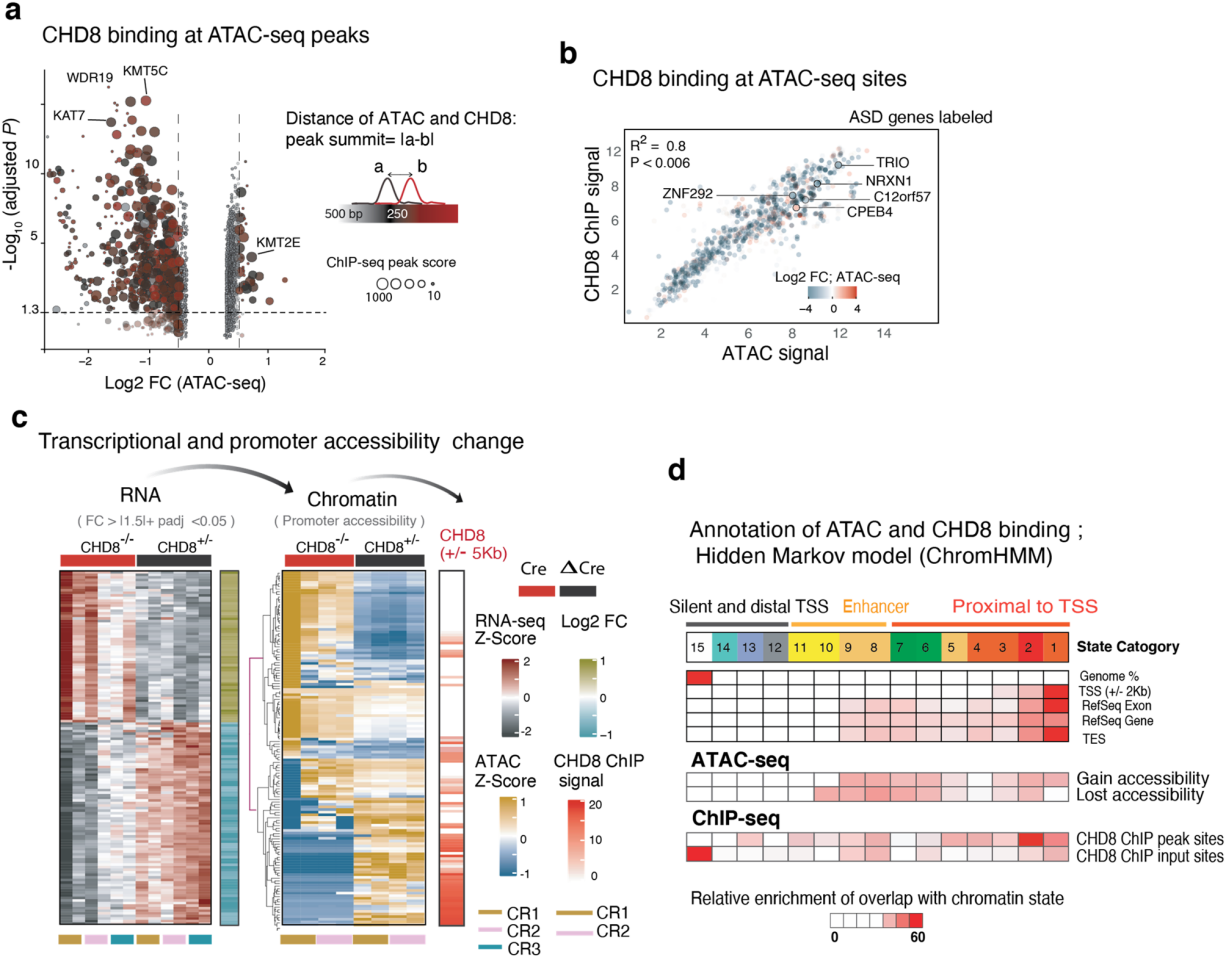
CHD8 induces accessible chromatin at its direct chromatin targets. (**a**) Volcano plot depicting each ATAC-seq peak as one dot. The color indicates the distance of the ATAC-seq peak summit from the CHD8 peak summit at the immediate vicinity, and the size reflects the CHD8 ChIP-seq peak score. (**b**) Linear regression of DESeq2 normalized CHD8 binding signal and DE-seq2 normalized ATAC-seq signal at the promoters of genes with change accessibility, and example ASD genes are labeled. The color indicates log2 change of the accessibility in CHD8 KO. (**c**) Analysis of chromatin modification enrichment with ChromHMM and the annotation of the genomic feature with transition probability for 15 state model uncovers the distribution and the relative enrichment of CHD8 binding and the ATAC-seq sites across all chromatin states in neurons (GEO accessions used for this analysis listed in Supplementary Table 1). (**d**) RNA-seq and ATAC-seq signal for 136 genes with a significant change in gene expression and ATAC-seq signal in KO. CHD8 binding signal sorted with the same order of the sites of the promoters at the respective heatmap.

The enrichment of an ETS motif was intriguing since we had already identified it among CHD8 binding targets in neurons. We therefore next investigated chromatin accessibility changes at CHD8 target sites in homozygous knockout neurons (Fig. 17a). Co-analysis of ATAC-seq and CHD8 ChIP-seq sites revealed that CHD8 binding is stronger at regions with increased chromatin accessibility (Fig. 18a). Conversely, in CHD8-KO cells, CHD8 target sites lost accessibility (Fig. 17b,d). Analysis of all overlapping ATAC-seq and CHD8 binding sites showed a significant loss of chromatin accessibility and transcription at CHD8 peak regions and associated target gene promoters (± 5 Kb); ASD-related genes were among the most highly changed genes. (Figs. 17c and 18b). Next, we analyzed CHD8 binding at the promoters of a distinct group of genes with strong differential expression and whose promoters change in chromatin accessibility upon CHD8 knockout. The results revealed that CHD8 binding is enriched at closing promoters in downregulating genes, providing further evidence that CHD8 may promote chromatin accessibility and induce gene expression (Fig. 18c).

### CHD8 targets harbor epigenetic signatures denoting actively transcribed genes

**Figure 19:**
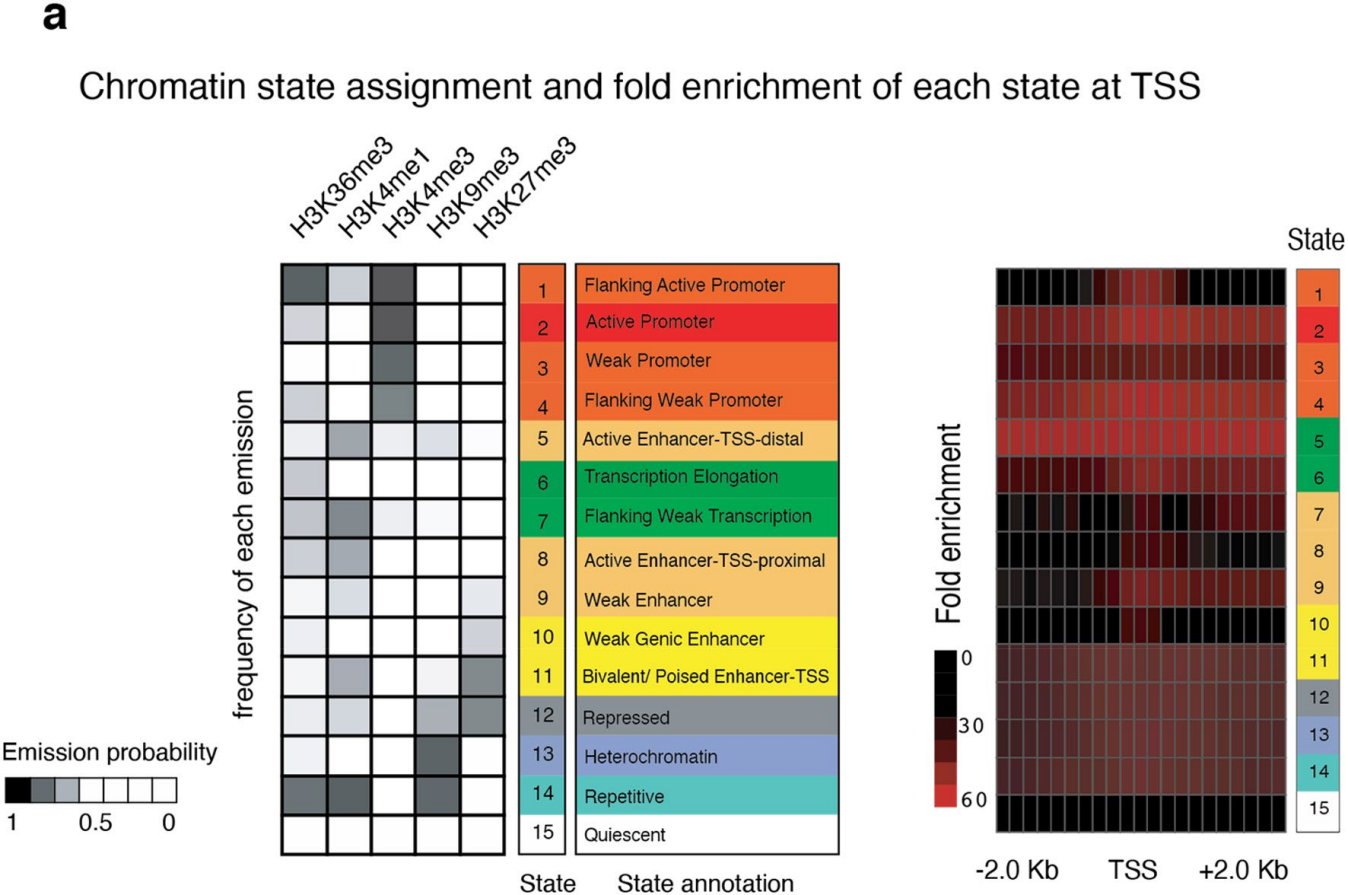
Analysis of chromatin state in neurons utilizing ChromHMM. (**a**) Chromatin state analysis**:** The right heatmap represents an enrichment of promoter-associated state at annotated TSS regions. The left plot shows enrichment of histone signal (ENCODE data from H9 derived neurons) for each annotated state as indicated by the emission probability value.

We next applied a multivariate hidden Markov model (HMM) to annotate the genome-wide chromatin state of CHD8 target regions using publicly available datasets for chromatin modifications from human H9-derived neurons^53,54^. First, we validated that our model accurately described the expected chromatin state at a group of actively transcribed promoters in neurons (n = 500) (Fig. 19a). Next, we analyzed the enrichment of CHD8 targets, including the sites of CHD8 binding and the ATAC-Seq peaks at annotated genome. Enrichment analysis revealed that CHD8 regulates chromatin accessibility at regions of the genome with an active chromatin state and with no preference for a distinct classification or mapping to a particular genomic annotation (e.g., promoters or enhancers). In contrast, CHD8 binding displayed a strong preference for proximal promoters (Fig. 18d).

### CHD8 affects chromatin accessibility ETS motif-containing sites

**Figure 20:**
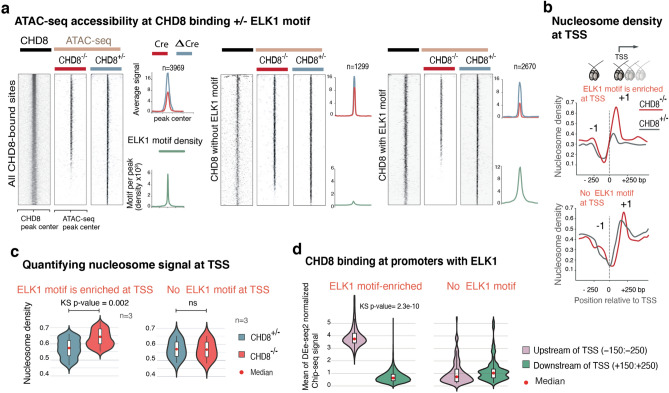
CHD8 and ELK1 cooperate to regulate chromatin accessibility. (**a**) Normalized CHD8 signal and ATAC-seq signal in *CHD8*-KO and the control samples plotted, and ELK1 motif density plotted as a green enrichment plot. Genome-wide ChIP-seq and ATAC-seq signal divided into three groups: sites with exclusive enrichment for ELK1 motif, sites without ELK1 motif, and the control sites, which are the randomly shuffled peak sets from control samples. For each group, we compared the ATAC signal from KO to the control sample. (**b**) Cross-correlation analysis of ATAC-seq signal with NucleoATAC to measure nucleosome density. The enrichment plot is a subset of the calculated nucleosome density signal taken from TSS with ± ELK1 (ETS) motif (motif occurrence > 1). (**c**) Violin plots compare normalized and averaged nucleosome density signal at 100 bp region around the position + 1 of TSS with the presence or absence of ELK1 motif. Statistical analysis of distribution comparison is calculated with the Kolmogorov Smirnov test. (**d**) CHD8 binding signal taken from 100 bp upstream and downstream of transcription start sites (TSS), with the presence or absence of ELK1 motif.

Next, we wondered whether our observed enrichment of ETS motifs at CHD8 targets may indicate a functional cooperation of ETS motif binding factors and CHD8. To this end, we plotted the ATAC seq peaks at CHD8 target regions with or without ETS motifs. We observed a much more pronounced loss of ATAC-seq signal at the ETS motif-containing sites than in ETS motif-free regions (Fig. 20a). Combined with ETS motif enrichments in CHD8 binding sites and CHD8-dependent ATAC-Seq sites, these results suggested a functional interaction between an ETS factor and CHD8 in regulating chromatin accessibility.

To further characterize ETS motif-dependent CHD8 activity, we implemented a cross-correlation analysis of the ATAC-seq signal to infer nucleosome density at transcriptional start sites (TSS) with ETS motifs but not at TSS lacking ETS motifs (Fig. 20b, c)^51^. We found the altered density of the + 1 nucleosome intriguing, prompting us to investigate the symmetry of CHD8 binding at promoters with and without ETS motifs. Indeed, the average CHD8 ChIP-seq signal (100 bp binned) upstream of promoters of ETS motif-containing genes actively transcribed in neurons was stronger than downstream of the promoters, on the other hand, sites not containing ETS motifs did not show such a relationship (Fig. 20d).

### CHD8 is recruited to ETS motif-containing sites by ELK1

**Figure 21:**
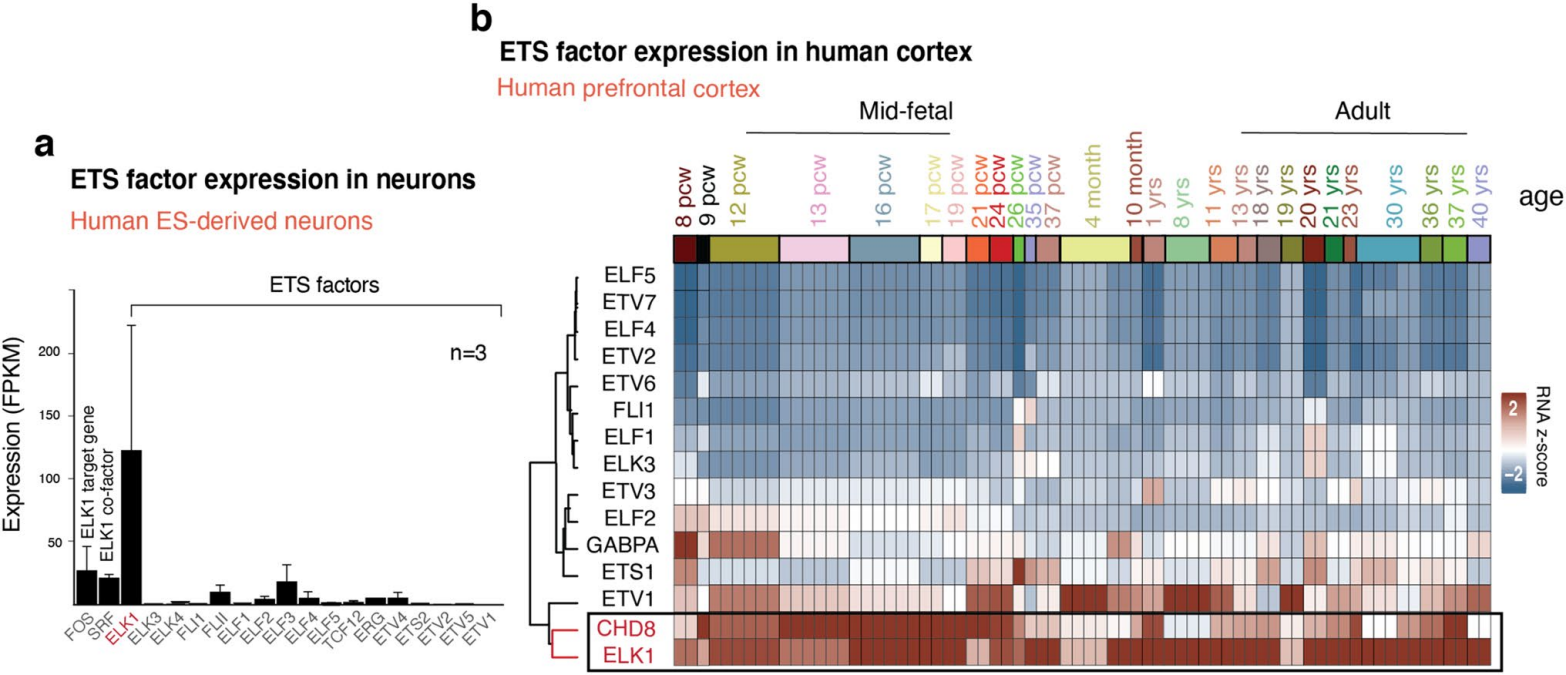
*ELK1* is the only highly expressed ETS genes in in-vitro derived human neurons and its expression cluster with CHD8 in human brain. (**a**) Average expression of ETS factors shows ELK1 is the only highly expressed ETS gene in differentiated human neurons (average FPKM values taken from wild type neurons). (**b**) Gene expression of *CHD8* and *ETS* factor analysis in human developing cortex and unsupervised clustering of expression levels (Alan brain data)^37^.

**Figure 22:**
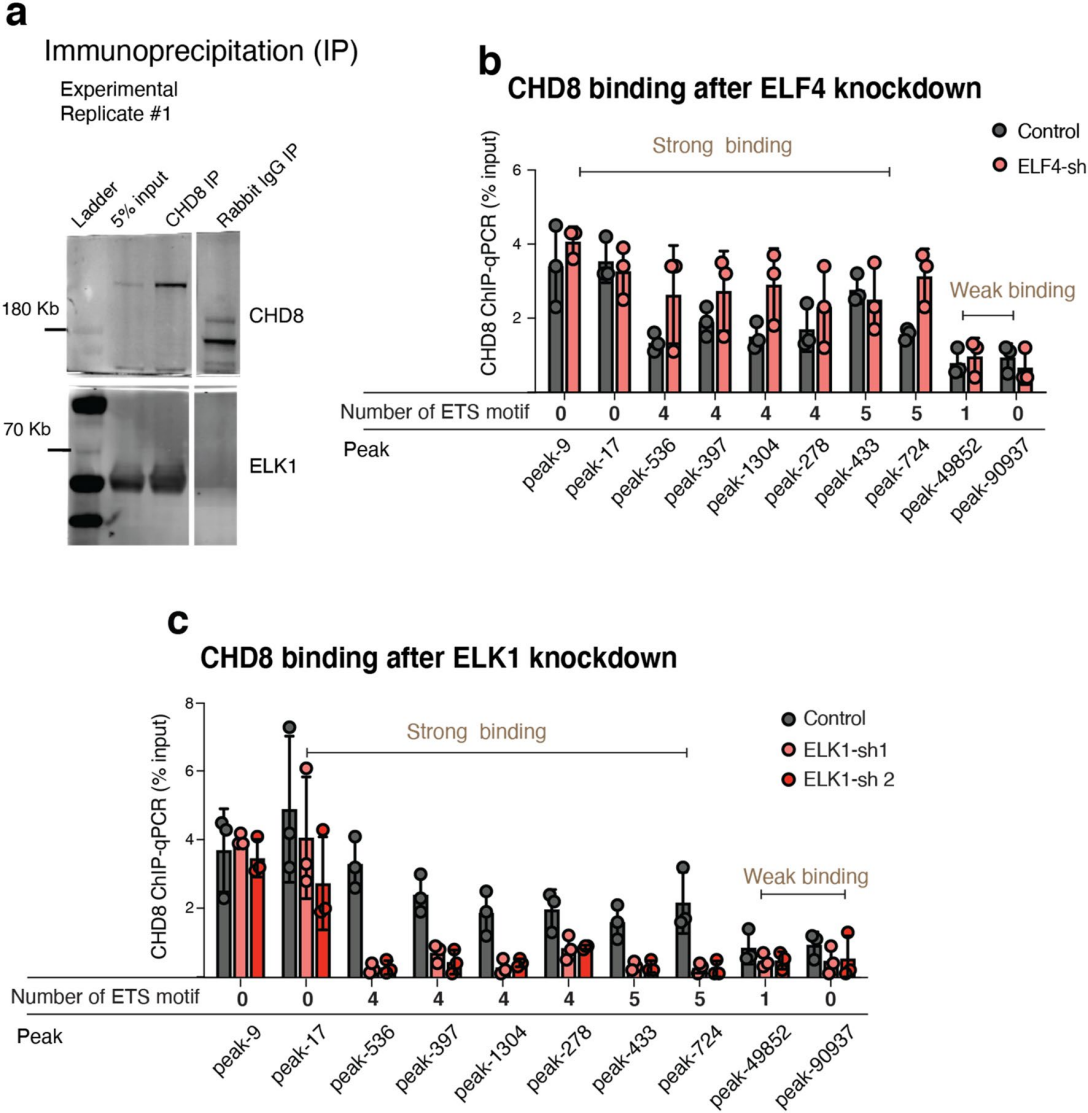
CHD8 co-immunoprecipitates with ELK1 in human neurons, and its chromatin binding perturbed in the absence of ELK1. (**a**) Pull-down assay for CHD8 and western blotting of CHD8 and ELK1 in human neurons (also see two other experimental replicates in Fig. 23a). (**b**) Knockdown of *ELF4* does not affect CHD8 binding on either of *ETS* or *YY1* motif sites, suggesting ELF4 does not influence chromatin binding of CHD8. (**c**) ChIP-qPCR for CHD8 binding after *ELK1* knockdown with two different hairpin RNAs (shRNAs). Control condition is an empty vector. The number of ETS motif at each peak region indicated beneath each peak. There was no change in CHD8 binding at sites without the *ETS* motif. See also Fig. 25c for validation of CHD8 peaks in CHD8-KO neurons.

**Figure 23:**
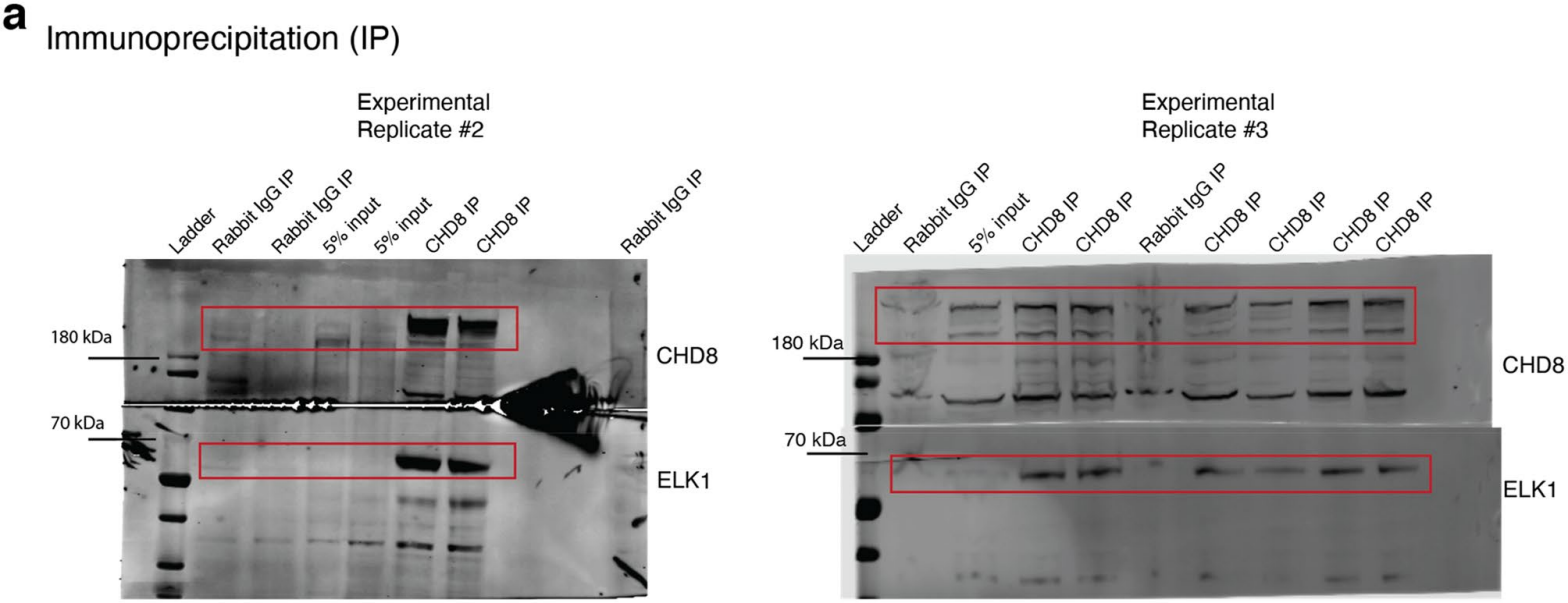
Pull down (IP) assay and detection of direct interaction between CHD8 and ELK1. (**a**) Two independent experimental replicates for IP of CHD8 and detection of ELK1 with western blot. Experimental replicate #1 shown in Fig. 24a.

To investigate which ETS factor may functionally interact with CHD8, we first turned to our gene expression data from wild-type neurons. We found that *ELK1* is the highest expressed ETS factor in human neurons (Fig. 21a)^37,55^. Next, we analyzed the expression of ETS factors in the human prefrontal cortex. Clustering analysis of single-cell RNA-seq data from the human prefrontal cortex revealed a correlative gene expression pattern between *ELK1* and *CHD8* but no other ETS factors (Fig. 21b). Furthermore, immunoprecipitation revealed that endogenous ELK1 co-immunoprecipitates with endogenous CHD8 in human neurons (one experimental replicate shown in Fig. 22a, and two replicates shown in Fig. 23a).

**Figure 24:**
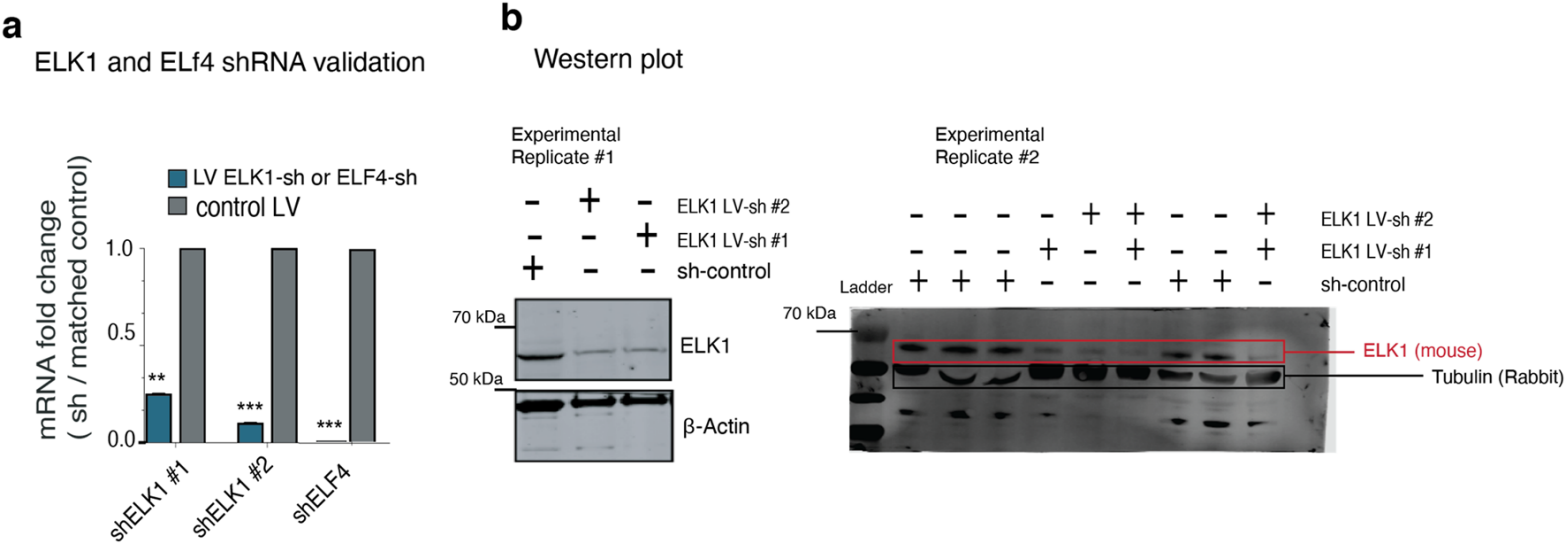
ELK1 RNA knockdown and measurements of protein level in neurons. (**a**) Bar graph shows knockdown of *ELK1* and *ELF4* genes with a short hairpin (shRNA) and measurement of mRNA. (**b**) Western blot shows knockdown of *ELK1* with two hairpins and the reduction of ELK1 protein in neurons (two independent experimental replicates).

**Figure 25:**
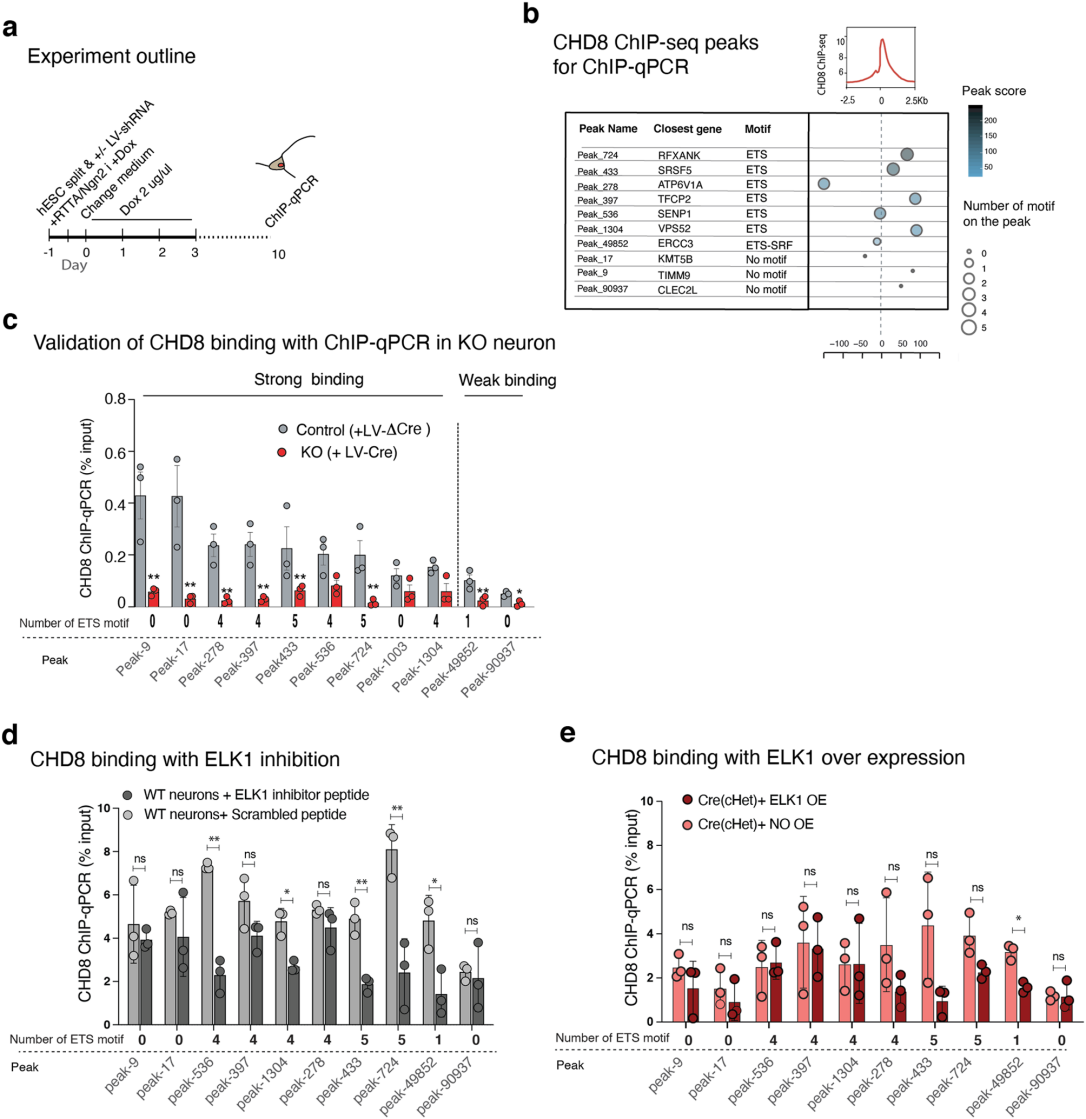
ELK1 perturbations and measurements of CHD8 binding to validated ChIP peaks. (**a**) Schematic depicts experiment outline and the timing of lentivirus-shRNA infection, dox treatment, and ChIP-qPCR experiment. (**b**) Table of selected CHD8 ChIP-seq peaks for ChIP-qPCR assessment; the number of motifs, peak score, and the location of each peak relative to TSS is shown. (**c**) ChIP-qPCR experiment to measure CHD8 binding on CHD8 ChIP-seq peaks in CHD8 knockout neurons. This experiment explicitly validates our ChIP-Seq results for CHD8 binding and provides good sites for downstream assays. (**d**) ChIP-qPCR for CHD8 binding with ELK1 inhibition in wild-type neurons. (**e**) ChIP-qPCR for CHD8 binding with ELK1 overexpression in CHD8 heterozygous knockout neurons (both red and pink barographs are CHD8 heterozygous knockout). The *p*-values are calculated with two-tailed T-test; *p < 0.01, **p < 0.001, ***p < 0.0001.

To examine the potential functional cooperativity between ELK1 and CHD8 in targeting chromatin, we sought to investigate whether ELK1 may affect CHD8 binding. To this end, we constructed lentiviral vectors with short hairpin RNA (shRNA) targeting *ELK1* and *ELF4* as a control. Quantitative qPCR and western blotting confirmed a robust decrease in mRNA and the protein after infecting the neurons with two hairpins against *ELK1* and one hairpin against *ELF4* (Fig. 24a,b). First, we validated the specificity of our selected ChIP-seq peaks for CHD8 binding by ChIP-qPCR in three independent pull-down experiments, which showed a complete absence of CHD8 binding in KO neurons (Fig. 25a–c). Next, we measured the binding of CHD8 at a series of CHD8 binding and ETS motif-enriched peak regions. Knockdown of the control factor (ELF4) did not change CHD8 binding at selected peaks (Fig. 22b). In contrast, CHD8 binding was specifically lost at ETS motif-containing CHD8 peaks upon ELK1 knockdown (Fig. 22c).

To further validate these findings, we treated neurons with a peptide inhibitor of ELK1^56,57^, and we observed similar results as we found with the shRNA experiments (Fig. 25d). Finally, overexpression of ELK1 did not significantly affect CHD8 binding, suggesting that ELK1 recruitment may be saturated or subject to posttranslational regulation such as phosphorylation that could not be captured by over expressing ELK1 (Fig. 25e). These results indicate that CHD8 and ELK1 interact and functionally cooperate in regulating chromatin accessibility and gene expression regulation in human neurons, and ELK1 might be guiding CHD8 to its motif sites at promoters.

## Discussion

The chromatin remodeling factor CHD8 is associated with developmental brain disorders, but its molecular function in mature human neurons is largely unknown^58^. To fill this knowledge gap, we report that CHD8 is responsible for maintaining an open chromatin configuration in promoters and overall transcriptional activation in human neurons. Our finding aligns with previous observations that showed CHD8 plays a role in the functional maturation of human oligodendrocyte precursors cells (OPCs) in a mechanism that involves recruiting histone methyltransferase complexes to target gene promoters^59^. We found that CHD8 regulates gene expression at active promoters enriched in the H3K4 methylation mark. Indeed, previous reports showed that CHD8 regulates Pol III-dependent promoters associated with H3K4 methylation^60^.

Our data also revealed functional cooperativity of *CHD8* and *ELK1* (the effector of MAPK/ERK) in chromatin regulation through a distinct model of directional activity oriented around the ELK1 motif.

Intriguingly, ELK1 plays a role in developing psychiatric disorders affecting the neural circuitry of the adult brain, and it is a prominent therapeutic target for treating depression and addiction^56,61,62^.

Finally, we revealed CHD8 regulates a distinct group of autism genes positively correlated in expression patterns in the developing human cortex, suggesting a conserved and developmentally regulated transcriptional connectivity between CHD8 and its targets. Our results indicate that MAPK/ERK/ELK1 may play a functional role in developing neuropsychiatric alterations caused by *CHD8* mutations. Modulation of specific aspects of this pathway, which regulate activity-dependent gene expression and synaptic plasticity, may represent a foundation to explore a therapeutic opportunity for functional interference with pathology induced by *CHD8* mutations^63,64^.

## Methods

### Resource table

Provided as a separate file in ‘Supplementary Table 1’.

### Software used to create figures

- Figures 1a,b, 2a,d, 3a and 11a generated with Adobe Illustrator 2022 (26.0.1). Figures 19a and 21a is ChromHMM^65^ figure, and labels are added using Adobe Illustrator 2022 (26.0.1) ‘type’ and ‘text tool.’
- Figure 4a`: Image credit is for Allen Institute for Brain Science.
- Figures 6b–e, 7a,b, 8a,b, 12a, 13a,c, 14a,b, 15b–d, 16b, 17b,c, 18a–c, 19b–d, 20a–c, 21b, 25b, prepared with ggplot2 version 3.3.5 and ComplexHeatmap, and R package. We used R version 4.1.3.
- Figure 25a was prepared in Adobe Illustrator, and the first author drew the cartoon. Figure 15a,
- Figure 12d, 24a prepared in Excel version 16.58. Figures 5e, 23a, 24b,c.
- Figures 5a–g, 10a–c, and 25c–e prepared in Prism version 9. Figures 13b, 22a.
- Figure 16a prepared using python program: deepTools (version 3.5.0).

### Cell culture

*CHD8*-KO human ES cells were generated from the human embryonic stem cell (ESC) line H9 (passage 50, WA09 WiCell Research Institute, Inc.) and an iPSC line from a male individual. Only cells with normal karyotype were used to generate conditional knockout cells and downstream analysis. Pluripotent stem cells were maintained in mTeSR1 (STEMCELL Technologies), and small-molecule Thiazovivin (5 µM) (STEMCELL Technologies) was applied to the medium before single-cell passaging. The conversion of PSC to induced neurons is described below according to our previously published protocol^36^.

### Lentivirus generation

Production of lentivirus was according to the previously described method^66^. In summary, about ten million the HEK293T cells were transfected with the packaging and the lentiviral vectors using Calcium-phosphate precipitation in a 10 cm petri dish. The cells were washed from transfection reagents the next day, and the supernatant was collected approximately 48 h after the transfection.

### Production of adeno-associated virus (AAV)

Recombinant adeno-associated virus (rAAV-DJ) is used to deliver the targeting vector to pluripotent stem cells. To produce rAAV, we co-transfected three plasmids: 25 µg of pAAV^67^, 25 µg of a helper plasmid (pAd5), and 20 µg of the capsid (AAV-DJ), into one T75 flask with 80% confluent HEK293T cells (ATCC) by calcium phosphate transfection method^67,68^. Two days after transfection, trypsin harvested cells for 10 min and lysed by three rounds of freeze and thawing in dry ice and a water bath (37 °C). The rAAV virus was collected from the supernatant by spinning the whole lysate and removing the pellet. The virus was aliquoted in small volumes to freeze at − 80 °C. Before using every 100 µl of supernatant, ten units of Benzonase endonuclease (EMD Chemical Inc, Merck 1.01695.002) were added at (37 °C) for 5 min to digest DNA from HEK cells; the capsid protects AAV DNA from digestion.

### Generation of human induced excitatory neurons (iN)

Human excitatory neurons differentiated from pluripotent stem cells by over-expression of lineage-specific transcription factor-Neurogenin 2 (Ngn2) as described before^36^. In summary, one day before conversion, we dissociated stem cells into single cells with Accutase (Innovative Cell Technologies) and seeded at ~ 40 K cells into one 24 well plate pre-coated with Matrigel (BD Biosciences) in a medium supplemented with Thiazovivin (5 µM) (STEMCELL Technologies) and doxycycline (2 mg/ml, Clontech). After 6 h, we infected the cells with lentivirus containing Ngn2, RTTA, and Cre recombinase or ΔCre (truncated form of Cre, which is not functional and is used as control). The next day, we replaced the medium with neuronal medium N2/DMEM/F12/NEAA (Invitrogen) containing doxycycline (2 mg/ml, Clontech). We kept the cells in this medium for five days, and on day six, we added ~ 10 K mouse glia cells into each 24 well and replaced the culture medium with a serum-containing medium. We analyzed the cultures approximately 3–5 weeks after induction. To generate homozygous knockout neurons, we infected the neurons with LV- Cre or ΔCre one day after induction of the Ngn2 transcription factor.

### Immunofluorescence (IF)

For immunofluorescence (IF) staining of cells (embryonic stem cells and iN cells), we fixed the cells using 4% paraformaldehyde (PFA) for 15 min at room temperature and permeabilized the cell membrane using 5% Triton for 1 h and then blocked the cells in a solution containing 1% BSA, 5% FBS and 1%Triton. The primary antibody was added to the same blocking buffer according to these dilutions: CHD8 antibody (Rabbit-Behtyl lab-A301-224A) used as 1:3,000, Synapsin1 antibody (Rabbit- Synaptic Systems-106002) used as 1:500, Homer1 (Rabbit-Synaptic Systems-160003) used as 1:500, HA (Rabbit-Sigma-H6908) used as 1:500, Map2 (Mouse-Sigma-M9942) used as 1:500,Tuj1 (Rabbit-Biolegened-802001) used as 1:500, ELK1 (Rabbit, Bethyl lab-A303-529A) used as 1:400 and incubated for O/N at 4 °C. DAPI was added as a 100 nM solution for 1 min. The secondary antibodies were made as 1:1,000 solutions and incubated for 1 h at room temperature.

### Western blotting and immunoprecipitation (IP)

Human stem cells and neurons were lysed with RIPA lysis buffer supplemented with 5 mM EDTA and protease inhibitor (Roche), for 5 min at room temperature and 10 min on ice. After the lysis, sample buffer (4 × Laemmli buffer containing 4% SDS, 10% 2-mecaptaneol, 20% glycerol, 0.004% 4-Bromophenol blue, 0.125 M Tris HCl, pH 6.8) was added, and the samples were either directly loaded on 4–12% SDS-PAGE gel or froze in -80 for further analysis. Approximately 20 to 30 µg of protein was separated on an SDS-PAGE gel for all the immunoblots. Antibodies used in this manuscript used with these dilutions: CHD8 antibody (Rabbit-Behtyl lab-A301-224A) used as 1:4,000, ELK1 (Rabbit, Bethyl lab-A303-529A) used as 1:1,000, HA (Rabbit-Sigma-H6908) used as 1:1,000, β-actin antibody (Rabbit, Abcam-ab8227) used as 1:20,000. Fluorescently labeled secondary antibodies visualized all blots on Odyssey CLx Infrared Imager with Odyssey software (LI-COR Biosciences). We lysed approximately 50 million human neurons grown in glia-free conditions for immunoprecipitation, differentiated from stem cells for ten days. We used five ugr anti-CHD8 antibodies to prepare protein G agarose beads blocked in serum-containing buffer overnight. We incubated cell lysate with an antibody-bound bead for three hours and washed the unbound fraction with house-made washing buffer containing 10% Triton -x-100, 0.5 M EGTA, 0.5 M PH8 EDTA, 5 M NaCl, and 1 M HEPES. We eluted the bound protein with elution buffer at 60 °C.

The blots are generally presented as full-length blot, and whenever there is cropping, it is due to cutting the membrane prior to hybridization with antibody. For heavily cropped blots we have provided additional technical replicates as full-length blot.

### RNA-sequencing

RNA was obtained from 3 weeks-old cultures of iN cells by adding Trizol LS (Thermo Fisher Scientific) directly into the cell culture well. Total 500 ng RNA processed for library preparation using “TruSeq” RNA sample preparation-V2 kit and “Ribo-Zero” rRNA removal kit (Illumina) according to manufacturer’s instruction. The sequencing ran on Illumina`s NextSeq 550 system with a 1 × 75-bp cycle run.

### RNA-seq data analysis

FastQ files were run on FastQC to obtain high-quality (trimmed and cleaned) reads. The reads were aligned to the human reference genome sequence (hg19) and assembled with TopHat/Bowtie (version 2.1.1)^69^ for transcriptome analysis. Since we generated the library from a mix of mouse and human RNA, the resulting reads were also from a mixture of both species. We, therefore, aligned our reads to the human genome with stringent criteria (zero mismatches allowed). The aligned sequences were randomly sampled and re-aligned to the other species' genome (the mouse, mm9 genome) to ensure that cross-species DNA alignment is not happening. Note that ~ 1% of the reads aligned to human and mouse genomes were discarded from SAM files with SAMtools^70^. The Refseq hg19 GTF file of transcriptome annotation was downloaded from Ensembl (https://uswest.ensembl.org/index.html) and used as a reference annotation file in the TopHat alignment run command to increase the speed and the sensitivity of alignments to splice junctions. Duplicate reads (from PCR step during library preparation) were removed with SAMtools. Pre-built indexes of bowtie were downloaded from the "Bowtie" webpage (http://bowtie-bio.sourceforge.net/tutorial.shtml). All SAMtools subcommands were used to convert SAM files to BAM files (Bindery Alignment Map). Additionally, SAMtools were used for indexing (to view the signal on the genome browser) and for sorting (necessary for downstream analysis). Cufflinks were used for transcript assembly and to estimate coding gene abundance (FPKM). To quantify transcripts across all the samples and obtain estimated counts for downstream analysis, we used HTSeq (htseq-count option)^71^. These raw counts were used as input for DESeq2 to perform differential expression analysis and to generate summarizing plots^72^.

*The single-cell RNA data of the human brain and the bulk RNA-seq of the developing human cortex* were obtained from the Allen Human Brain Atlas, and the Image credit in Fig. 1 (with some modification) is the Allen Institute^37^. We used Seurat V3 with default parameters for each function^73^.

### ChIP-seq and data analysis

ChIP-seq was performed with modifications from a published protocol^74^. In summary, ten confluent 10 cm plates of iN cells (approximately 10 × 10^6^ neurons in total) 10 days after differentiation were used for chromatin extraction. Cultures were crosslinked with 1% Formaldehyde (Sigma) for 10 min at RT. Glycine (125 mM) was added to quench and terminate the cross-linking reaction, and after washing with PBS, cells were scraped off the dishes and collected into a 50 mL tube. DNA samples were subjected to sonication to obtain an average fragment size of 200–600 bp, using Covaris (S220-Focused Ultrasonicator). After sonication, the pellets were cleared from debris by centrifugation at 4 °C. The supernatant was collected for further analysis of DNA fragment size (column-purified DNA ran in 2% agarose gel to determine the size) and for DNA/protein concentration analysis. For input calculation, approximately 0.5% of cross-linked chromatin was separated and saved before the addition of IP antibodies. For immunoprecipitation (IP), 1.5 µg anti-CHD8 or anti-HA antibody was added to ChIP buffer (RIPA buffer supplemented with protease inhibitors, PMSF, and 5 mM EDTA) left to rotate O/N at 4 °C. At the same time, protein G agarose beads (Active Motif) were washed and blocked with 5% BSA in ChIP buffer and left to rotate O/N at 4 °C. The next day, the antibody-bound chromatin was added to protein G and spun 5 h in 4 °C. The immunoprecipitated material was washed, and the IP material was eluted from beads with elution buffer (50 mM NaCl, Tris–HCl; pH 7.5) by vortexing at 37 °C for 30 min. The eluted DNA was separated from beads by spinning. For reverse cross-linking, the IP and input material were incubated at 65 °C/shaking, RNaseA (10 ug/ul), and 5 M NaCl plus proteinase K (20 ug/ul). DNA was purified on a column (Zymo Research) and processed for library preparation. NEBNext ChIP-seq library prep kit was used for library preparation. Sequencing was performed on Illumina`s NextSeq 550 system with a 1 × 75-bp cycle run. We obtained 18 to 20 million total reads per sample in one sequencing run.

### ATAC-seq experiment

We followed the Pi-ATAC-seq protocol to transpose homozygous knockout and control neurons^75^. The cells were fixed in culture for 5 min with 1% PFA, detached from the plate with EDTA, and stained for GFP, allowing us to sort the Cre-GFP positive cells. After that, the transposition proceeded as standard ATAC-seq protocol with slight modification (extra step of reverse cross-linking performed overnight in 65C^o^). Note that for the heterozygous knockout and wild-type transposition, we followed the original ATAC-seq protocol in which un-fixed nuclei are permeabilized and subjected to transposition^76^.

### ATAC-seq, ChIP-seq data analysis

For ChIP-seq and ATAC-seq, ENCODE ChIP-seq pipline2 was used to obtain significant peaks^41,53^. For motif discovery, we used HOMER (v4.10) (http://homer.ucsd.edu/homer/). For clustering analysis, we used Cluster 3.0^77^. Heatmaps were generated using the R program: ComplexHeatmap^78^. For ontology analysis, we used DAVID analytical tool^79^. To obtain estimated counts within the region of interest in the ATAC-seq experiment we used FeatureCounts- a general-purpose read count tool from Rsubread package^80^ and a custom GTF file with the coordinates of the overlapping ATAC-seq peak in all the samples used as input for the program. For library normalization and differential accessibility analysis, we used DESeq2^72^. Differential accessible sites (opening and closing regions) were manually examined in UCSC Genome Browser with the 2019 update (http://genome.ucsc.edu). For enrichment analysis and generating normalized heatmaps and signal intensity plots, we used “deepTools,” using normalized bigWig files as input and bin size of 10 for almost all the heatmaps^81^.

### *NucleoATAC* analysis

We employed python package and the below code to obtain nucleosome position and occupancy (occ) from ATAC-seq files:

***** Calling nucleosomes

nucleoatac run –bed input-chip.bed –bam corresponding-input-chip.bam –fasta hg19.fa –out occ-out-file

***** To obtain nucleosome position and normalized signal

nucleoatac nuc –bed Encode-outout.narrowPeak.gz –vmat out.VMat –bam ATAC.bam –out nucleoatac-nuc-files

***** To convert bedgraph to bigwig file observed in genome browser or generate normalized density plot with the output we employed

./bedGraphToBigWig nucleoatac_signal.smooth.bedgraph hg19.chrom.sizes nucleatac- _signal.smooth.bigwig

### ChromHMM analysis

We used the ChromHMM algorithm to characterize neuronal chromatin state and the functional chromatin domains at CHD8 targets. We obtained histone mark ChIP-seq data of H9 derived neurons from ENCODE portal^65^. The histone signals are binarized across the genome to build a multivariate hidden Markov model and learn histone modification's combinatorial and spatial pattern at CHD8 target regions^41^.

An example code for running one file:

***** Binarizing input file:

java -mx1200M -jar ChromHMM.jar BinarizeBed -b 500 -peaks hg19.chromsizes cellmarkfiletable.txt/output-binarized

***** Learn model

java -mx1200M -jar ChromHMM.jar LearnModel -b 500 -init random /output-binarized input leanrmodel 10 hg19

### ChIP quantitative PCR (ChIP-qPCR) experiment

Total 5–10 ng ChIP DNA and the input were used to perform a quantitative PCR experiment and measure the enrichment levels. All primers used are listed in

“ChIP-seq-peaks.xlsx” file (attached to GSE141085), along with the relevant information, including the closest gene and the number of the motif on the peak. Three independent technical replicates (independent IP experiments) were used for qPCR analysis for each peak site. We normalized the ChIP signal over the input signal, less than 0.5% for total IP material. Analysis of qPCR experiment performed on the light Cycler 480II (Roche).

### RNA extraction and RT-qPCR experiment for gene expression

For RT-qPCR and RNA-seq experiments, we applied similar RNA isolation methods: neurons differentiated on mouse glia cells for ~ 3 weeks were washed in PBS and then lysed with TRIzol added directly to the plate. RNA was purified with the ZYMO RESEARCH- Direct-zol kit. Human-specific primers were used for amplification of the desired RNA.

### Analysis of dendritic arborizations

Neuronal cultures were fixed at approximately three weeks after transgene induction with 4% PFA for 15 min. The primary and secondary antibodies dilutions are according to our method in the “Immunofluorescence experiment.” For morphological analysis and tracing neurites, we used the MetaMorph^82^ software, and for synaptic puncta analysis and other general image processing, we used the java program ImageJ and the relevant modules, including CellProfiler 3.0^83^.

### AAV-mediated gene targeting

For the generation of conditional CHD8 heterozygous knockout cell line, we designed a donor vector for homologous recombination that carries two homology arms around the exon 4 of the CHD8 gene and included two loxP sequences in the same direction for frameshifting mutation. A positive selection cassette (neomycin expression to confer resistance to Geneticin) was included for purifying clones that carry the integrated donor cassette. The selection cassette contained a splice acceptor (SA) and a sequence for internal ribosomal entry site (IRES) attached to the Neomycin resistance gene (NEO) and a polyadenylation (PA) signal. The NEO resistant clones were used for screening PCR to verify the correct insertion of the targeting vector in the locus (Fig. 2a–c). The PCR primers are designed to cover the region from outside the homology arm (primers # 1 and #4) to inside the cassette.

The drug resistance cassette was flanked with FRT sequence and later removed by transient expression of FlpE recombinase. For HA-FLAG tagging of the CHD8 gene, the tags were inserted into the C-terminus region in the frame before the stop codon of Exon 38, together with the Neomycin resistance gene (see Fig. 2a).

After infection of ES cells with recombinant AAV (rAAV-DJ) carrying ITR flanked targeting vectors, we selected the cells with Geneticin antibiotic (Gibco) for ten days or until single colonies were obtained. The resistant colonies expanded, and genomic DNA was extracted for downstream analysis.

The selected subclones from ES and iPS cells were cultured on MEF and expanded to extract genomic DNA and perform screening PCR. The number of picked colonies was around a hundred and fifty, and the number of positive colonies (positive PCR band for screening PCR assay from both sides of the homology arms) was five. We confirmed the correct genotype for three subclones: C1, C2, and C3.

### CRISPR-Cas9 knockout

To generate CHD8 knockout cells, a frameshift mutation is introduced to non floxed allele by Nucleofection of 5 ugr Cas9 plasmid (lentiCRISPR v2-addgene # 52961) and two ugr guide RNA that cover exon four (spacer sequence: tagcaccatcactcctgtag) transfected into five to six million ES cells with the use of nucleofection method. One day after transfection, the cell culture media changed with a cell medium containing puromycin to select the cells that contain the Cas9-Puro lentiviral construct. The resistant cells were left to grow to form colonies and later picked for DNA extraction and Sanger sequencing. The number of selected colonies was fifty-five. The number of positive colonies (indel occurring in the non-floxed allele) was six, and three subclones out of six carried frameshift mutation in the non-floxed allele. We confirmed the correct genotype for three of the subclones: CR1 (2 bp deletion; ES cell line), CR2 (10 bp insertion; ES cell line), and CR3 (7 bp deletion; iPSC line).

### Electrophysiology

Electrophysiological recordings in cultured iN cells were performed in the whole-cell configuration as described previously^36,84^. Patch pipettes were pulled from borosilicate glass capillary tubes (Warner Instruments) using a PC-10 pipette puller (Narishige). The resistance of pipettes filled with intracellular solution varied between 2 and 4 MOhm. The standard bath solution contained (in mM): 140 NaCl, 5 KCl.

2 CaCl_2_, 2 MgCl_2_, 10 HEPES–NaOH pH 7.4, and 10 glucose; 300–305 mosm/l. Excitatory postsynaptic currents (EPSCs) were pharmacologically isolated with picrotoxin (50 µM) and recorded at − 70 mV holding potential in voltage-clamp mode with a pipette solution containing (in mM): 135 CsCl, 10 HEPES-CsOH pH 7.2, 5 EGTA, 4 MgATP, 0.3 Na_4_GTP, and 5 QX-314; 295–300 mosm/l. Evoked EPSCs were triggered by a 0.5-ms current (100 µA) injection through a local extracellular electrode (FHC concentric bipolar electrode, Catalogue number CBAEC75) placed 100–150 µm from the soma of neurons recorded. The frequency, duration, and magnitude of the extracellular stimulus were controlled with a Model 2100 Isolated Pulse Stimulator (A-M Systems, Inc.) synchronized with the Clampex 9 data acquisition software (Molecular Devices). Spontaneous miniature EPSCs (mEPSCs) were monitored in the presence of tetrodotoxin (TTX, 1 µM). The mEPSC events were analyzed with Clampfit 9.02 (Molecular Devices) using the template matching search and a minimum threshold of 5pA, and each event was visually inspected for inclusion or rejection. Intrinsic action potential (AP) firing properties of iN cells were recorded in current-clamp mode using a pipette solution that contained (in mM): 123 K-gluconate, 10 KCl, 7 NaCl, 1 MgCl_2_, 10 HEPES–KOH pH 7.2, 1 EGTA, 0.1 CaCl_2_, 1.5 MgATP, 0.2 Na_4_GTP and 4 glucose; 295–300 mosm/l. First, minimal currents were introduced to hold membrane potential around − 70 mV, next, the increasing amount of currents (from − 10 to + 60 pA, five pA increments) were injected for 1 s in a stepwise manner to elicit action potentials. Input resistance (R_in_) was calculated as the slope of the linear fit of the current–voltage plot generated from a series of small subthreshold current injections. To determine whole-cell membrane capacitance, square wave voltage stimulation was used to produce a pair of decaying exponential current transients that were each analyzed using a least-squares fit technique (Clampfit 9.02). Neuronal excitability recordings were performed using a standard bath solution supplemented with 20 µM CNQX, 50 µM AP5, and 50 µM PTX to block all possible glutamatergic (AMPAR- and NMDAR-mediated) as well as GABAergic synaptic transmission. Drugs were applied to the bath solutions before all recordings. Data were digitized at 10 kHz with a 2 kHz low-pass filter using a Multiclamp 700A amplifier (Molecular Devices). For all electrophysiological experiments, the experimenter was blind to the condition/genotype of the cultures analyzed. All experiments were performed at room temperature.

### Quantifications and statistical analysis

All data are shown as means + -SEM and from a minimum of three biological replicates (independent differentiations). GraphPad Prism and R were used for statistical analysis and calculations of significance.

## Supplementary Information

Supplementary Information 1.

## Data Availability

The raw sequencing files are deposited with the Gene Expression Omnibus (NCBI) (GEO accession number: GSE141085). The list of Encode data used in this study is listed in “the ChIP-seq-peaks.xlsx” file (attached to GSE141085).
